# Multifactorial profiling of epigenetic landscapes at single-cell resolution using MulTI-Tag

**DOI:** 10.1038/s41587-022-01522-9

**Published:** 2022-10-31

**Authors:** Michael P. Meers, Geneva Llagas, Derek H. Janssens, Christine A. Codomo, Steven Henikoff

**Affiliations:** 1 Basic Sciences Division, Fred Hutchinson Cancer Research Center, Seattle, WA USA; 2 Howard Hughes Medical Institute, Chevy Chase, MD USA; 3 Present Address: Department of Genetics, Washington University School of Medicine in St. Louis, St. Louis, MO USA

**Keywords:** Chromatin analysis, Epigenetic memory

## Abstract

Chromatin profiling at locus resolution uncovers gene regulatory features that define cell types and developmental trajectories, but it remains challenging to map and compare different chromatin-associated proteins in the same sample. Here we describe Multiple Target Identification by Tagmentation (MulTI-Tag), an antibody barcoding approach for profiling multiple chromatin features simultaneously in single cells. We optimized MulTI-Tag to retain high sensitivity and specificity, and we demonstrate detection of up to three histone modifications in the same cell: H3K27me3, H3K4me1/2 and H3K36me3. We apply MulTI-Tag to resolve distinct cell types and developmental trajectories; to distinguish unique, coordinated patterns of active and repressive element regulatory usage associated with differentiation outcomes; and to uncover associations between histone marks. Multifactorial epigenetic profiling holds promise for comprehensively characterizing cell-specific gene regulatory landscapes in development and disease.

## Abstract (web summary)

A scalable barcoding method measures multiple chromatin-associated proteins in single cells.

## Main

Single-cell sequencing methods for ascertaining cell-type-associated molecular characteristics by profiling the transcriptome^1–3^, proteome^4–6^, methylome^7,8^ and accessible chromatin landscape^9,10^, in isolation or in ‘multimodal’ combinations^11–15^, have advanced rapidly in recent years. More recently, methods for profiling the genomic localizations of proteins associated with the epigenome, including Tn5 transposase-based Cleavage Under Targets & Tagmentation (CUT&Tag)^16,17^, have been adapted for single-cell profiling. The combinatorial nature of epigenome protein binding and localization^18–20^ presents the intriguing possibility that a method for profiling multiple epigenome characteristics at once could derive important information about cell-type-specific epigenome patterns at specific loci. However, precise, scalable methods for profiling multiple epigenome targets simultaneously in the same assay are still lacking. Motivated by this gap, and with the knowledge that CUT&Tag profiles chromatin proteins in single cells at high signal-to-noise ratio^16^, we developed MulTI-Tag, a method for physical association of a chromatin protein-targeting antibody with an identifying adapter barcode added during tagmentation that could be used to deconvolute epigenome targets directly in sequencing.

## Results

**Fig. 1:**
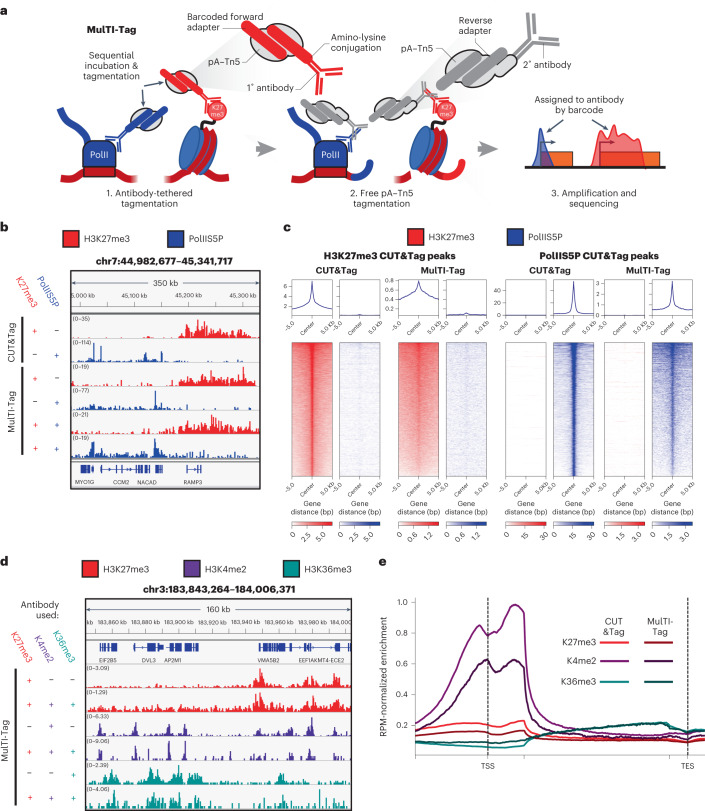
MulTI-Tag directly identifies user-defined chromatin targets in the same cells. **a**, Schematic describing the MulTI-Tag methodology. (1) Antibody–oligonucleotide conjugates are used to physically associate forward-adapter barcodes with targets and are loaded directly into pA–Tn5 transposomes for sequential binding and tagmentation. (2) pA–Tn5 loaded exclusively with reverse adapters are used for a secondary CUT&Tag step to efficiently introduce the reverse adapter to conjugate-bound loci. (3) Target-specific profiles are distinguished by barcode identity in sequencing. **b**, Genome browser screenshot showing individual CUT&Tag profiles for H3K27me3 (first row) and RNA PolIIS5P (second row) in comparison with MulTI-Tag profiles for the same targets probed individually in different cells (third and fourth rows) or sequentially in the same cells (fifth and sixth rows). **c**, Heat maps describing the enrichment of H3K27me3 (red) or RNA PolIIS5P (blue) signal from sequential MulTI-Tag profiles at CUT&Tag-defined H3K27me3 peaks (left) or RNA PolIIS5P peaks (right). **d**, Genome browser screenshot showing H3K27me3 (red), H3K4me2 (purple) and H3K36me3 (teal) MulTI-Tag signal from experiments in H1 hESCs using an individual antibody (rows 1, 3 and 5) or all three antibodies in sequence (rows 2, 4 and 6). **e**, Normalized CUT&Tag (light colors) and MulTI-Tag (dark colors) enrichment of H3K27me3, H3K4me2 and H3K36me3 across genes in H1 hESCs. RPM, reads per million.

**Extended Data Fig. 1:**
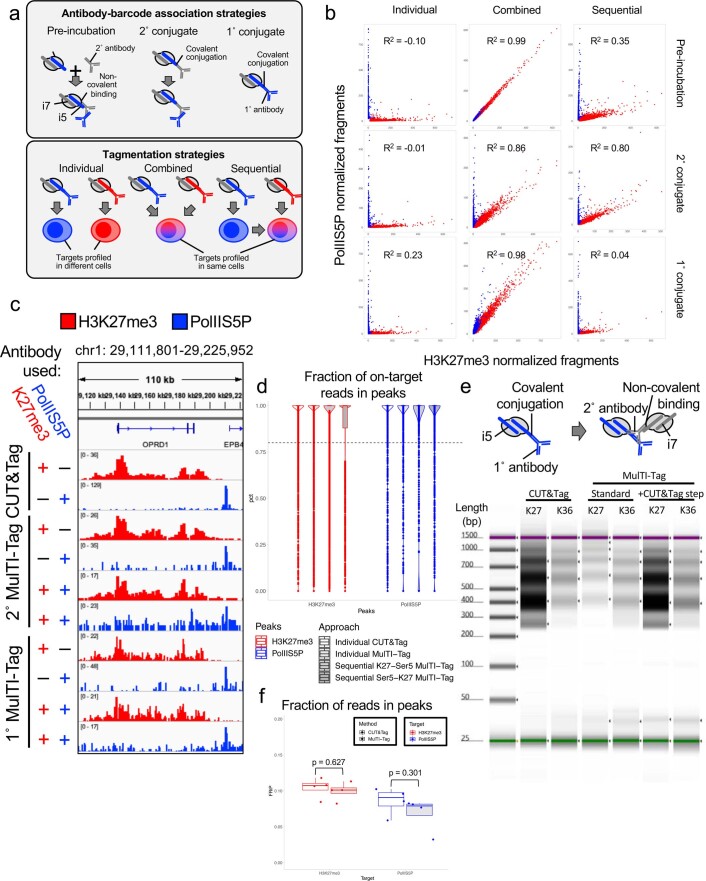
Design and validation of MulTI-Tag. a) Schematic of protocol variations tested for distinguishing CUT&Tag targets by sequencing barcode. Top: Approaches for pairing barcodes with antibodies, either by pre-incubation of barcoded pA-Tn5 with a secondary antibody (‘Pre-incubation’, left), or covalent conjugation of barcode-containing adapters to secondary (‘2° conjugate’, center) or primary (‘1° conjugate’, right) antibodies. Bottom: Approaches for tagmenting multiple targets, either in separate cells (‘Individual’, left), in the same cells simultaneously (‘Combined’, center), or in the same cells sequentially (‘Sequential’, right). b) Scatterplots describing the enrichment of H3K27me3 (X-axis) and PolIIS5P (Y-axis) in H3K27me3 (red points) or PolIIS5P (blue points) peaks for combinations of experimental conditions described in 2a. Pearson’s R^2^ of all data points is denoted for each of the nine protocol conditions. c) Genome browser screenshot showing individual CUT&Tag profiles for H3K27me3 (first row) and RNA PolIIS5P (second) in comparison with MulTI-Tag profiles for the same targets probed individually in different cells (third and fourth rows secondary conjugate MulTI-Tag; seventh and eighth rows primary conjugate MulTI-Tag) or sequentially in the same cells (fifth and sixth rows secondary conjugate MulTI-Tag; ninth and tenth rows primary conjugate MulTI-Tag). d) Violin plot describing distribution of fraction of on-target reads in peaks, defined as the percentage of reads corresponding to the same target for which the peak was called, from CUT&Tag (columns 1 and 5), single-antibody MulTI-Tag (2 and 6), sequential MulTI-Tag with H3K27me3 tagmented first (3 and 7), or sequential MulTI-Tag with PolIIS5P tagmented first (4 and 8). All calculations are based on peaks called from H3K27me3 (red) and PolIIS5P (blue) ENCODE ChIP-seq data. e) Top: Schematic of MulTI-Tag with additional CUT&Tag step, in which 1° antibody conjugates are loaded into pA-Tn5 along with free i5 adapter (left), and secondary antibody and pA-Tn5 loaded only with i7 adapter are added before tagmentation (right). Bottom: TapeStation HSD1000 trace describing DNA size and enrichment from libraries produced from CUT&Tag (lanes 1 and 2), "standard" MulTI-Tag with conjugate-only tagmentation (3 and 4), or MulTI-Tag with a secondary CUT&Tag step as described in methods (5 and 6), targeting H3K27me3 (1, 3, and 5) or H3K36me3 (2, 4, and 6) in K562 cells. f) Boxplots describing Fraction of Reads in Peaks (FRiP) score, defined as the fraction of a single target’s total unique reads mapping to peaks called for that target, calculated for H3K27me3 (red) or PolIIS5P (blue) ENCODE ChIP-seq peaks for four biological replicates each from CUT&Tag or sequential MulTI-Tag. Chi-square test p-values are denoted above comparisons.

Using antibodies against mutually exclusive Histone H3 lysine 27 trimethylation (H3K27me3) and RNA polymerase II phosphorylated at serine 5 of the C-terminal domain (PolIIS5P) in human K562 chronic myelogenous leukemia cells as controls, we systematically tested a variety of protocol conditions for antibody–barcode association with the goal of optimizing both assay efficiency and fidelity of target identification (Extended Data Fig. 1a). In contrast with previous reports^21^, we found that both pre-incubation of barcoded protein A-Tn5 (pA–Tn5) complexes and combined incubation and tagmentation of all antibodies simultaneously resulted in high levels of spurious cross-enrichment between targets (Extended Data Fig. 1b,c), leading us to use adapter-conjugated antibodies loaded into pA–Tn5 to tagment multiple targets in sequence. We also found that tagmenting in sequence beginning with the target predicted to be less abundant (PolIIS5P in this case) modestly reduced off-target read assignment (Extended Data Fig. 1d). We further found that primary antibody conjugates resulted in superior target distinction versus secondary antibody conjugates (Extended Data Fig. 1b,c) but also variable data quality, likely owing to fewer pA–Tn5 complexes accumulating per target locus in the absence of a secondary antibody. To overcome this obstacle, we (1) loaded pA–Tn5 onto 1° antibody-conjugated i5 forward adapters; (2) tagmented target chromatin in sequence; and (3) added a secondary antibody followed by pA–Tn5 loaded with i7 reverse adapters and carried out a final tagmentation step (Fig. 1a). This resulted in libraries that were as robust as matched CUT&Tag experiments, particularly for H3K27me3 (Extended Data Fig. 1e). We dubbed this combined approach MulTI-Tag (Fig. 1a). MulTI-Tag profiles for each of H3K27me3 and PolIIS5P profiled in sequence were highly accurate for on-target peaks as defined by ENCODE chromatin immunoprecipitation followed by sequencing (ChIP-seq) (Fig. 1b,c) and had similar specificity of enrichment to CUT&Tag as measured by fraction of reads in peaks (Extended Data Fig. 1f), indicating that MulTI-Tag recapitulates target enrichment without cross-contamination that may confound downstream analysis.

**Extended Data Fig. 2:**
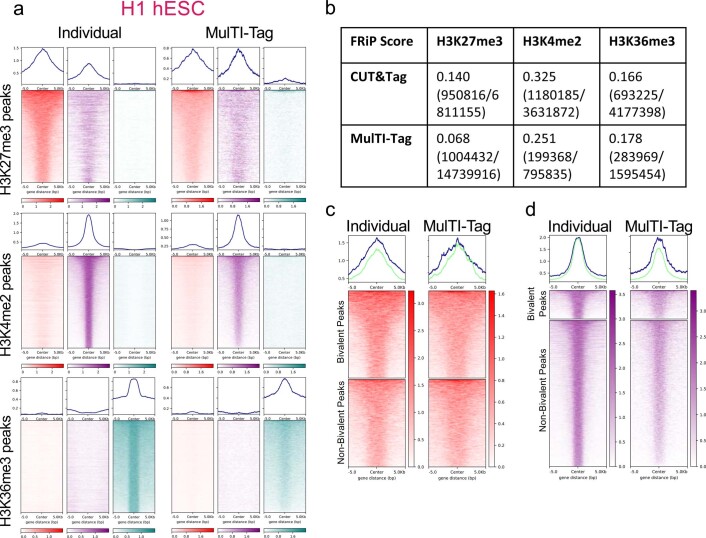
MulTI-Tag profiling in bulk H1 hESCs. a) Heatmaps describing the enrichment of H3K27me3 (red), H3K4me2 (purple), or H3K36me3 (teal) signal from H1 cell MulTI-Tag profiles using single antibodies (left) or three antibodies sequentially (right) in H3K27me3 (top), H3K4me2 (middle), or H3K36me3 (bottom) peaks. b) Table describing Fraction of Reads in Peaks (FRiP) score in ENCODE ChIP-seq peaks for H3K27me3, H3K4me2, and H3K36me3 for CUT&Tag and MulTI-Tag experiments in H1 cells. c) Heatmaps describing comparative enrichment of H3K27me3 in bivalent (top) vs. non-bivalent (bottom) enriched regions in CUT&Tag (left) or MulTI-Tag (right) experiments. d) Heatmaps describing the same as c) for H3K4me2.

In H1 human embryonic stem cells (hESCs), we simultaneously profiled three targets that represent distinct waypoints during developmental gene expression: H3K27me3, enriched in developmentally regulated heterochromatin^22,23^; H3K4me2, enriched at active enhancers and promoters^24^; and H3K36me3, co-transcriptionally catalyzed during transcription elongation^25,26^ (Fig. 1d,e). In comparison with control experiments in which each of the three targets was profiled individually, MulTI-Tag retains similar accuracy of target-specific enrichment in peaks (Extended Data Fig. 2a) and efficiency of signal over background (Extended Data Fig. 2b). Moreover, both control and MulTI-Tag experiments exhibit characteristic patterns of enrichment for each mark, including H3K4me2 at promoters, H3K36me3 in gene bodies and H3K27me3 across both (Fig. 1e). Of note, we observed regions with overlap between H3K27me3 and H3K4me2 for both CUT&Tag and MulTI-Tag samples consistent with known ‘bivalent’ chromatin in hESCs^27^. The enrichment of these regions in our MulTI-Tag was similar to standard CUT&Tag, indicating that tagmenting targets in sequence does not preclude detection of expected co-enrichment of two targets at the same loci (Extended Data Fig. 2c,d).

**Fig. 2:**
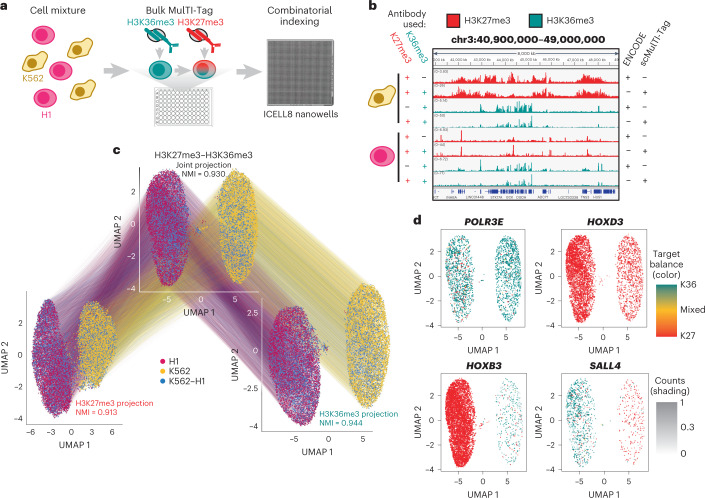
MulTI-Tag in single cells. **a**, Schematic describing single-cell MulTI-Tag experiments. H1 hESCs (fuschia) and K562 cells (gold) were profiled separately or in a mixture of the two cell types in bulk, and then cells were dispensed into nanowells on a Takara ICELL8 microfluidic device for combinatorial barcoding via amplification. **b**, Genome browser screenshot showing aggregated single-cell MulTI-Tag data (rows 2, 4, 6 and 8) in comparison with ENCODE ChIP-seq data (rows 1, 3, 5 and 7) profiling H3K27me3 (rows 1, 2, 5 and 6) and H3K36me3 (rows 3, 4, 7 and 8) in K562 (rows 1–4) and H1 (rows 5–8) cells. All single-cell MulTI-Tag data are from cells co-profiled with H3K27me3 and H3K36me3. **c**, Connected UMAP plots for single-cell MulTI-Tag data from H1 and K562 cells. Projections based on H3K27me3 (left), H3K36me3 (right) or a WNN integration of H3K27me3 and H3K36me3 data (center) are shown. NMI of cell type cluster accuracy is denoted for each projection. Lines are connected between points that represent the same single cell in different projections. **d**, WNN UMAP projections with MulTI-Tag enrichment scores plotted for *POLR3E* (top left), *HOXD3* (top right), *HOXB3* (bottom left) and *SALL4* (bottom right). The balance of enrichment between H3K36me3 and H3K27me3 in each cell is denoted by color, and the total normalized counts in each cell are denoted by the transparency shading.

**Extended Data Fig. 3:**
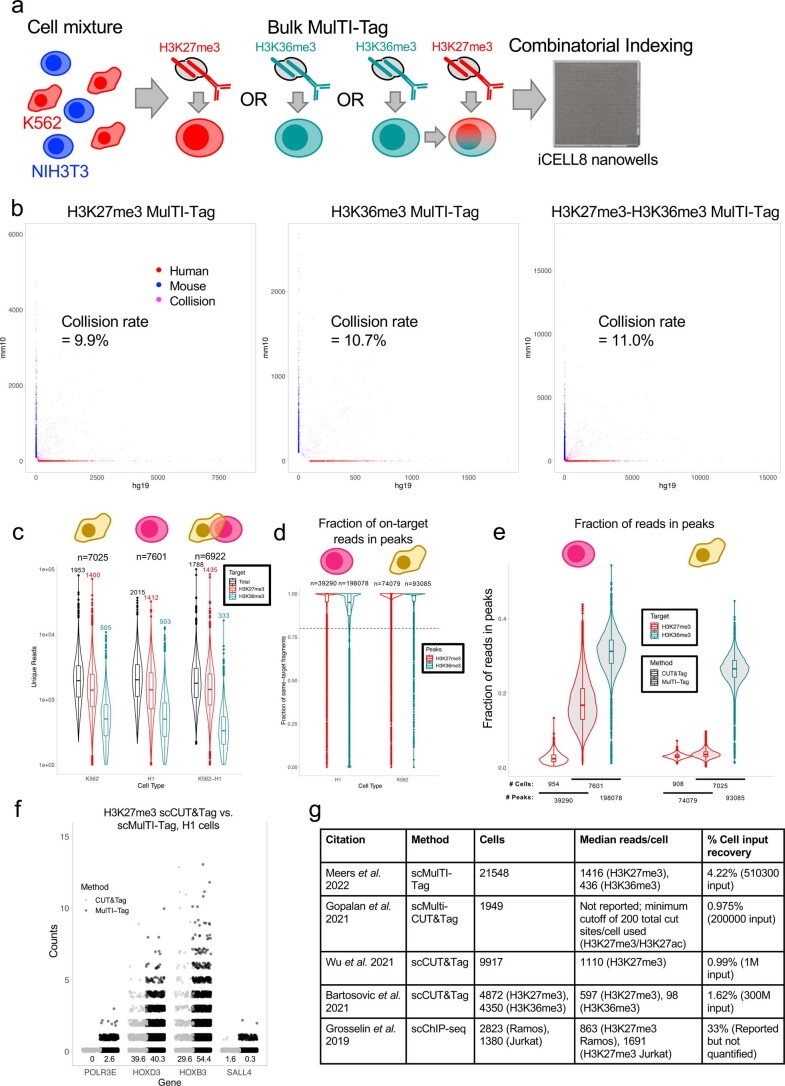
Combinatorial indexing for single-cell MulTI-Tag. a) Schematic describing single cell MulTI-Tag species mixing experiments. Human K562 cells (red) and mouse NIH3T3 cells (blue) were mixed and profiled in bulk, then cells were dispensed into nanowells on a Takara ICELL8 microfluidic device for combinatorial barcoding via amplification. b) Barnyard plots describing the number of unique fragments exclusively mapping to the hg19 genome build (X-axis) vs. mm10 (Y-axis) in all cells with greater than 100 unique reads for each of the denoted experiments. Points are colored by the cell identity as human (red; > 90% of unique reads mapping to hg19), mouse (blue; >90% mapping to mm10), or mixed (magenta; < 90% mapping to either), and collision rate, defined as the percentage of cells classified as ‘mixed’, is denoted for each experiment. c) Violin plots describing distributions of unique reads per cell in K562 cells (left), H1 cells (center), or the K562-H1 cell mixed population (right). Median values for total unique reads (black), H3K27me3 unique reads (red), or H3K36me3 unique reads (teal) are displayed at the top of each violin. Number of cells described is displayed at top of each cell type group. d) Violin plot describing distribution of fraction of on-target reads in peaks, defined as the percentage of reads corresponding to the same target for which the ENCODE ChIP-seq peak was called, in H3K27me3 (red) and H3K36me3 (teal) peaks from single cell MulTI-Tag in H1 cells (left) and K562 cells (right). Number of peaks is displayed above each violin. e) Violin plots describing Fraction of Reads in Peaks (FRiP) score in ENCODE ChIP-seq peaks for H3K27me3 (red) or H3K36me3 (teal) data from single cell CUT&Tag (white) or sequential single cell MulTI-Tag (grey). Number of cells described and number of peaks used is displayed below each violin. f) Jittered scatterplot describing the number of counts mapping to each single cell within each of the indicated genes in single cell CUT&Tag^29^ (black) vs. single cell MulTI-Tag (grey). The percentage of cells with non-zero counts for each locus and assay are denoted at the bottom. g) Table describing comparative metrics for MulTI-Tag (this study) in comparison with scMulti-CUT&Tag^21^, scCUT&Tag^28,29^, and scChIP-seq^32^.

Given the successful adaptation of CUT&Tag for single-cell profiling^16,28–30^, we sought to use MulTI-Tag for single-cell molecular characterization (Fig. 2a). To do so, we adapted the Takara ICELL8 microfluidic system for unique single-cell barcoding via combinatorial indexing (Fig. 2a and Methods). In a pilot combinatorial indexing MulTI-Tag experiment profiling H3K27me3 and H3K36me3 either individually or in combination in a mixture of human K562 cells and mouse NIH3T3 cells, we calculated cross-species collision rates as 9.9% (231/2,334, H3K27me3), 10.7% (173/1,623, H3K36me3) and 11.0% (358/3,262, H3K27me3–H3K36me3) of cells yielding <90% of reads from a single species (Extended Data Fig. 3a,b). These statistics are similar to the same metrics reported for combinatorial indexing-based assay for transposase-accessible chromatin with sequencing (ATAC-seq) (7–12%^10,31^). To confirm that MulTI-Tag could be used to distinguish a mixture of cells originating from the same species, we jointly profiled H3K27me3 and H3K36me3 in K562 cells, H1 hESCs and a mixture of the two cell types, yielding 21,548 cells (7,025 K562, 7,601 H1 and 6,922 Mixed) containing at least 100 unique H3K27me3 and 100 unique H3K36me3 reads (Fig. 2b and Extended Data Fig. 3c). For most peaks defined by ENCODE ChIP-seq (91.4% and 92.4% for H3K27me3 in H1 and K562 cells; 84.9% and 94.8% for H3K36me3 in H1 and K562 cells), more than 80% of fragments corresponded to the expected target (Extended Data Fig. 3d,e). Moreover, MulTI-Tag uniformity of coverage at representative loci (Extended Data Fig. 3f), cell recovery from input, and library complexity as measured by unique reads per cell were similar or superior to analogous published methods for single-cell chromatin profiling^21,28,29,32^ (Extended Data Fig. 3g).

**Extended Data Fig. 4:**
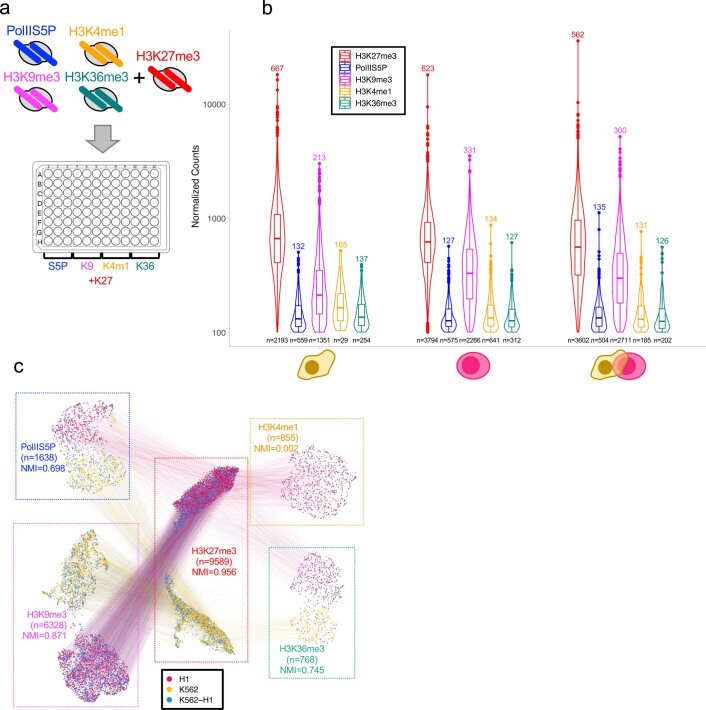
MulTI-Tag across diverse target combinations. a) Schematic describing single cell MulTI-Tag profiling different combinations of targets in the same combinatorial indexing experiment. One of four targets (PolIIS5P, H3K9me3, H3K4me1, or H3K36me3) was tagmented in sequence with H3K27me3 in bulk, then arrayed in a 96 well plate as displayed for i7 tagmentation (Methods). b) Violin plots describing distributions of unique reads per cell in K562 cells (left), H1 cells (center), or the K562-H1 cell mixed population (right) for the experiments described in a). Median values for H3K27me3 unique reads (red), PolIIS5P unique reads (blue), H3K9me3 unique reads (magenta), H3K4me1 unique reads (orange), or H3K36me3 unique reads (teal) are displayed at the top of each violin. Number of cells described for each cell type-target combination is displayed at the bottom of each violin. c) Connected UMAP plots for single cell MulTI-Tag data from experiments described in a). Projections based on H3K27me3 (center), PolIIS5P (top left), H3K9me3 (bottom left), H3K4me1 (top right), or H3K36me3 (bottom right) are shown. Total cells represented and normalized mutual information (NMI) of cell type cluster accuracy are denoted for each projection. Lines are connected between points that represent the same single cell in different projections.

We used uniform manifold approximation and projection (UMAP)^33,34^ to project single-cell data into low-dimensional space based on enriched features defined for H3K27me3, H3K36me3 or a combination of both based on weighted nearest neighbor (WNN) integration^35^ and clustered the resulting projections (Fig. 2c). Using our known cell type labels to calculate cluster normalized mutual information (NMI) on a scale of 0 (no cell type distinction by cluster) to 1 (perfect cell type distinction by cluster), H3K27me3 (0.913), H3K36me3 (0.944) and H3K27me3–H3K36me3 combined (0.930) were all highly proficient in cluster distinction (Fig. 2c). Additionally, 99.1% (6,383/6,443) of ‘Mixed’ cells occupied non-ambiguous clusters defined nearly exclusively by either H1 or K562 cells (Fig. 2c). Constitutively expressed (*POLR3E*) or silenced (*HOXD3*) genes exhibited cluster non-specific enrichment of H3K36me3 and H3K27me3, respectively, and genes expressed exclusively in K562 (*HOXB3*) or H1 (*SALL4*) cells were enriched for H3K36me3 in the cell-specific cluster versus H3K27me3 in the other (Fig. 2d). To further demonstrate the flexibility of target combinations possible with MulTI-Tag, we profiled K562, H1 and K562–H1 Mixed cells in three additional target pair combinations (H3K27me3–PolIIS5P, H3K27me3–H3K9me3 and H3K27me3–H3K4me1) (Extended Data Fig. 4a,b). All individual marks distinguished cell types with high efficiency with the exception of H3K4me1, likely owing to the fact that only 27 K562 cells were analyzed for H3K4me1 enrichment after quality control filtering (Extended Data Fig. 4c). In all, these results show that MulTI-Tag can use enrichment of multiple targets to distinguish mixtures of cell types.

**Fig. 3:**
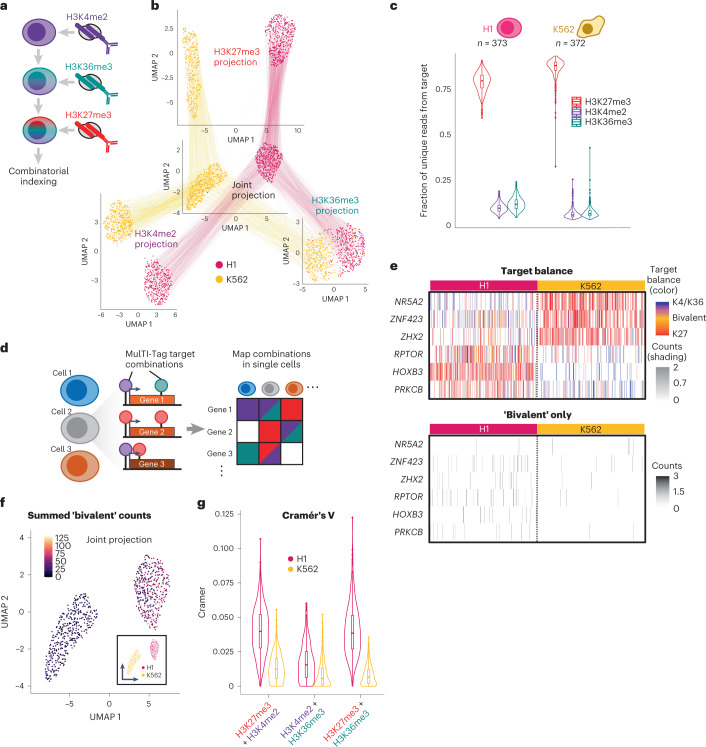
Coordinated multifactorial analysis in the same cells using MulTI-Tag. **a**, Schematic describing a three-antibody MulTI-Tag experiment. **b**, Connected UMAP plots for single-cell MulTI-Tag data from H1 and K562 cells. Projections based on H3K27me3 (top), H3K4me2 (left), H3K36me3 (right) or a WNN integration of H3K27me3, H3K4me2 and H3K36me3 data (center) are shown. Lines are connected between points that represent the same single cell in different projections. **c**, Violin plots describing the distribution of the proportions of MulTI-Tag H3K27me3 (red), H3K4me2 (purple) or H3K36me3 (teal) unique reads out of total unique reads in individual H1 (left) or K562 (right) cells. **d**, Schematic describing coordinated multifactorial analysis strategy for MulTI-Tag. Genes in individual cells are analyzed for the enrichment of all MulTI-tag targets, and gene–cell target combinations are mapped onto a matrix for clustering and further analysis. **e**, Top: heat map describing co-occurrence of MulTI-tag targets in six genes of interest in each of 373 H1 cells and 372 K562 cells. The balance of enrichment between H3K4me2/H3K36me3 and H3K27me3 in each cell is denoted by color, and the total normalized counts in each cell are denoted by the transparency shading. Bottom: Instances of ‘bivalent’ enrichment of H3K27me3 and H3K4me2 or H3K36me3 in the same gene in the same cell are highlighted, with color reflecting normalized counts. **f**, WNN UMAP projection with cells colored by the sum of all counts occurring in a ‘bivalent’ context (that is, H3K27me3 and H3K4me2/H3K36me3 enrichment in the same gene). **g**, Violin plots describing calculated Cramér’s V of association between target combinations listed at bottom in individual H1 (fuschia, *n* = 373) or K562 (gold, *n* = 372) cells.

**Extended Data Fig. 5:**
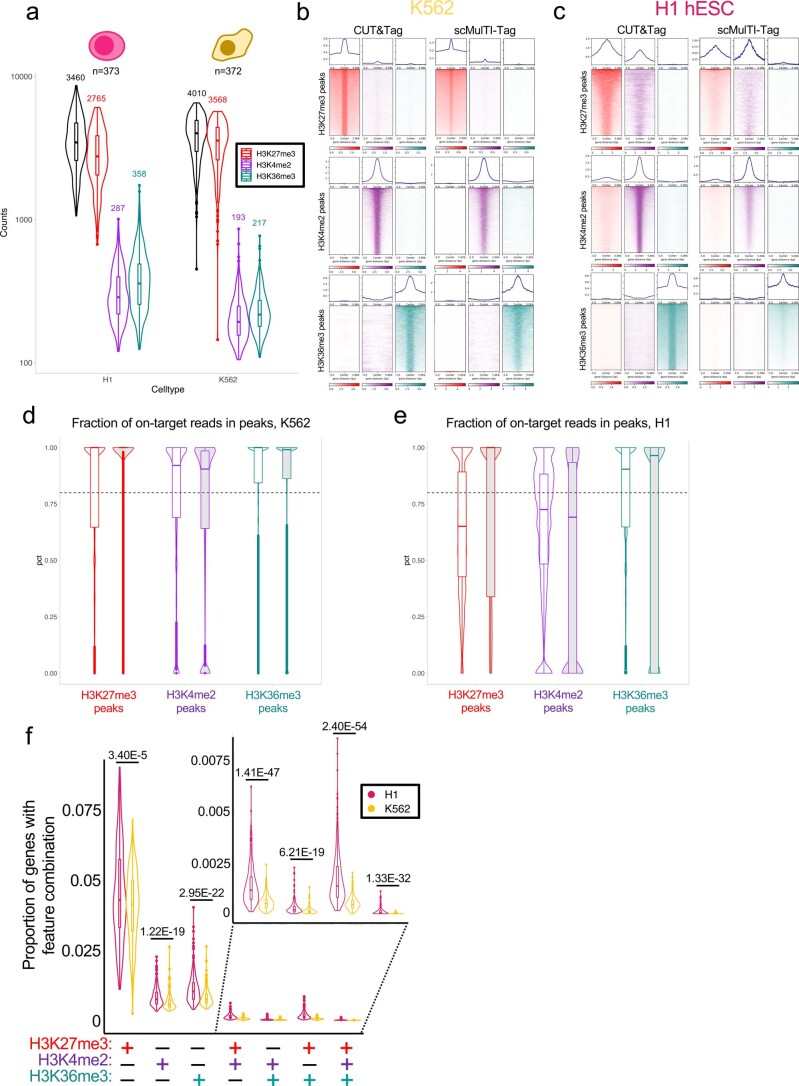
Cross-target analysis in scMulTI-Tag. a) Violin plots describing distributions of unique reads per cell in H1 cells (left) or K562 cells (right) for experiments described in Fig. 3. Median total unique reads (black), H3K27me3 unique reads (red), H3K4me2 unique reads (purple), or H3K36me3 unique reads (teal) are displayed at the top of each violin. Number of cells described is displayed at top of each cell type group. b) Heatmaps describing the enrichment of H3K27me3 (red), H3K4me2 (purple), or H3K36me3 (teal) signal from K562 cell profiles using single antibodies in bulk MulTI-Tag (left) or three antibodies sequentially in aggregate single cell MulTI-Tag (right) in H3K27me3 (top), H3K4me2 (middle), or H3K36me3 (bottom) peaks as called from bulk MulTI-Tag data. c) Heatmaps describing the same as b) for H1 hESCs. d) Violin plots describing the distribution of the fraction of on-target reads in peaks, defined as the percentage of reads corresponding to the same target for which the ENCODE ChIP-seq peak was called, in H3K27me3 (red, n = 74079), H3K4me2 (purple, n = 65388), and H3K36me3 (teal, n = 93085) peaks from bulk individual MulTI-Tag (white) vs. sequential single cell MulTI-Tag (grey) in K562 cells. e) Violin plots describing the same as d) for H1 hESCs (H3K27me3 n = 39290, H3K4me2 n = 119250, H3K36me3 n = 198078). f) Violin plots describing the distributions of proportions of each co-occurrence state as described below the plot in individual H1 (fuschia, n = 373) or K562 (gold, n = 372) cells, with points denoting individual cell values. The last four co-occurrence states are rescaled and inset at top right; p-values derived from two-sided student’s t-test comparing distributions between cell types are listed above violins (not corrected for multiple hypothesis testing).

**Extended Data Fig. 6:**
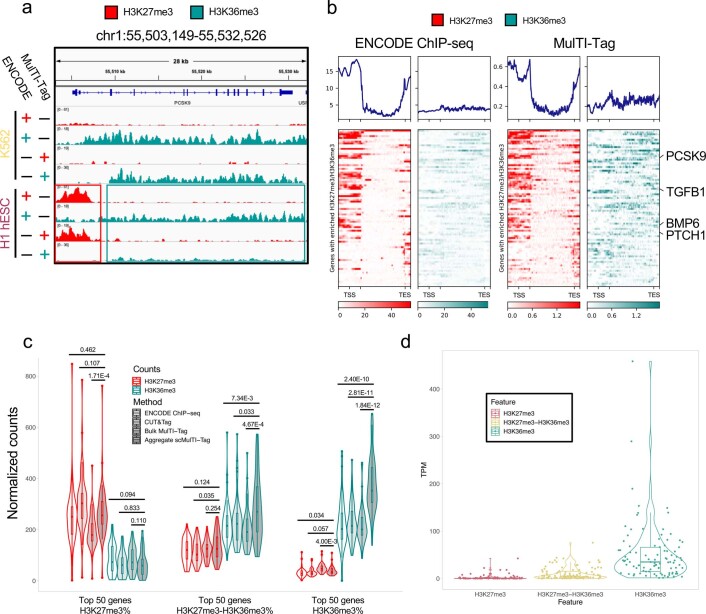
Verification of H3K27me3-H3K36me3 co-enrichment in MulTI-Tag. a) Genome browser screenshot showing H3K27me3 (red) and H3K36me3 (teal) enrichment from ENCODE ChIP-seq (rows 1, 2, 5, and 6) or bulk MulTI-Tag (rows 3, 4, 7, and 8) in K562 cells (rows 1-4) or H1 hESCs (rows 5-8) at the PCSK9 gene. Colored boxes indicate co-enrichment of H3K27me3 and H3K36me3 in the same gene in H1 hESCs. b) Heatmaps describing the enrichment of H3K27me3 (red) and H3K36me3 (teal) signal from ENCODE ChIP-seq (left) or bulk MulTI-Tag (right) in H1 hESCs in 86 genes for which 1) a MulTI-Tag H3K27me3 peak overlapped a 2 kb window surrounding the TSS, and 2) a MulTI-Tag H3K36me3 peak overlapped the gene body. Selected genes of interest, including those involved in metabolic and developmental signaling, are highlighted at right. c) Violin plots describing the number of normalized counts for H3K27me3 (red) and H3K36me3 (teal) mapping to the top 100 genes as classified by the percentage of single H1 hESCs enriched with H3K27me3 (left), H3K36me3 (right), or co-enriched for H3K27me3 and H3K36me3 (center) in the genes in question. ENCODE ChIP-seq (white), CUT&Tag (light grey), bulk MulTI-Tag (medium grey) and aggregate single cell MulTI-Tag (dark grey) counts are displayed for each category. P-values derived from student’s t-tests are listed above violins. d) Violin plots describing ENCODE RNA-seq counts mapping to the top 100 genes as classified by the percentage of single H1 hESCs enriched with H3K27me3 (left), H3K36me3 (right), or co-enriched for H3K27me3 and H3K36me3 (center) in the genes in question.

Because MulTI-Tag uses barcoding to define fragments originating from specific targets, we can directly ascertain and quantify relative target abundances and instances of their co-occurrence at the same loci in single cells. To establish methods for cross-mark analysis in single cells, we co-profiled the aforementioned transcription-associated marks (H3K27me3–H3K4me2–H3K36me3) by MulTI-Tag in single H1 and K562 cells with high target specificity (Fig. 3a,b and Extended Data Fig. 5a–e). When we calculated the percentage of unique reads originating from each of the three profiled targets in each single cell, we found that H3K27me3 represented the vast majority (89.4% and 80.0% in K562 cells and H1 cells) of unique reads (Fig. 3c). This is consistent with previously reported mass spectrometry^36^ and single-molecule imaging^37^ quantification of H3K27me3 versus H3K4me2 species and with a reported higher abundance of H3K27me3 in differentiated versus pluripotent cells^38^. By mapping fragments from any target in H1 and K562 cells onto genes in a window from 1 kilobase (kb) upstream of the transcription start site (TSS) to the gene terminus, we found notable instances of genes that show co-enrichment of distinct targets in the same single cells, including H3K4me2 and/or H3K36me3 enrichment in *NR5A2* linked with H3K27me3 enrichment in *HOXB3* in the same H1 cells and vice-versa in K562 cells (Fig. 3e). We were also able to classify genes by the frequency with which they were singly or co-enriched with specific targets in an individual cell. H1 hESCs had a higher frequency of most co-enriched target combinations than K562 cells (Extended Data Fig. 5f), including ‘bivalent’ H3K27me3–H3K4me2 co-enrichment in the same gene in individual cells^27^ (Fig. 3e,f). We used Cramér’s V (ref. ^39^) to quantify the degree of co-enrichment between each pair of targets in the same genes in the same single cells, and we confirmed that H1 cells had a higher degree of co-enrichment between H3K27me3 and H3K4me2 than K562 cells (Fig. 3g). Curiously, the same was true for association between H3K27me3 and H3K36me3, despite previous observations that H3K27me3 and H3K36me3 appear to be antagonistic in vitro and in vivo^40,41^ (Fig. 3g). Nevertheless, in CUT&Tag, in bulk MulTI-Tag and in previously published ENCODE ChIP-seq data from H1 hESCs, we were similarly able to detect co-occurrence of H3K27me3 at the 5′ ends and H3K36me3 at the 3′ ends of several genes, concomitant with their low expression as quantified by ENCODE RNA sequencing (RNA-seq) data (Extended Data Fig. 6a–d). Together, these results shed light on patterns of chromatin enrichment at single-cell, single-locus resolution.

**Fig. 4:**
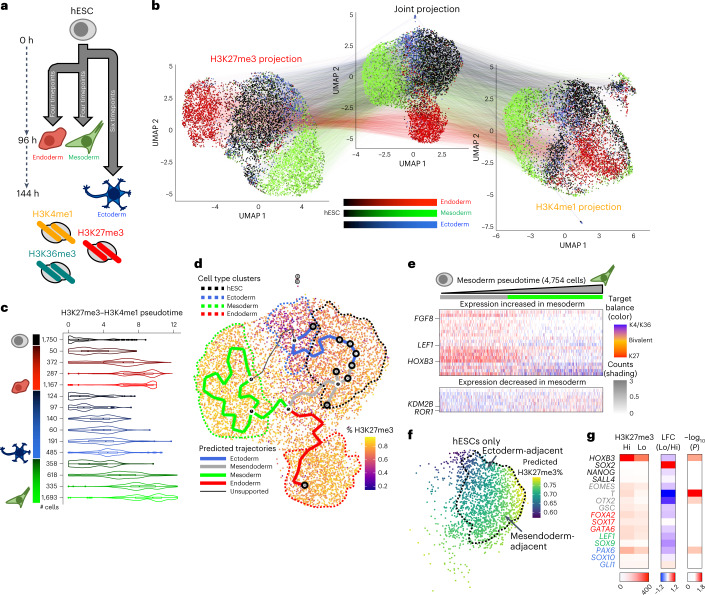
MulTI-Tag profiling of continuous developmental trajectories. **a**, Schematic describing differentiation of H1 hESCs (black) into three germ layers—Ectoderm (blue shading), Endoderm (red shading) and Mesoderm (green shading)—followed by MulTI-Tag profiling of H3K27me3, H3K4me1 and H3K36me3. **b**, Connected UMAP plots for single-cell MulTI-Tag data from H1 hESCs differentiated to three germ layers. Projections based on H3K27me3 (left), H3K36me3 (right) or a WNN integration of H3K27me3 and H3K36me3 data (center) are shown. Lines are connected between points that represent the same single cell in different projections. **c**, Violin plot showing the distribution of inferred pseudotimes derived from a WNN integration of H3K27me3 and H3K4me1 data for each cell type profiled. Number of cells profiled for each cell type is denoted at left. **d**, WNN UMAP projection colored by percent H3K27me3 as a proportion of total unique reads in each single cell. User-defined cell type clusters are denoted by dashed lines, and computationally derived pseudotemporal trajectories are denoted by solid lines and user-classified by color. **e**, Heat map describing co-occurrence of MulTI-tag targets in selected genes of interest whose RNA-seq expression increases (top) or decreases (bottom) during differentiation from hESC to mesoderm in 4,754 single cells classified as hESC or different stages of differentiated mesoderm. Heat maps are sorted left to right by increasing pseudotime in the mesendoderm/mesoderm trajectory. The balance of enrichment between H3K4me1/H3K36me3 and H3K27me3 in each cell is denoted by color, and the total normalized counts in each cell are denoted by the transparency shading. **f**, hESCs plotted to the WNN UMAP projection and colored by predicted H3K27me3 percent as a proportion of total unique reads (Methods). hESCs adjacent to the ectoderm trajectory or the mesendoderm trajectory are denoted by arrows. **g**, Heat maps denoting H3K27me3 enrichment in ‘high-H3K27me3’ and ‘low-H3K27me3’ hESCs (left); log fold change (LFC) in enrichment (center); and −log_10_(*P* value) of differential enrichment (right) for select genes colored by their function in hESCs (black), mesendoderm (gray), endoderm (red), mesoderm (green) or ectoderm (blue).

**Extended Data Fig. 7:**
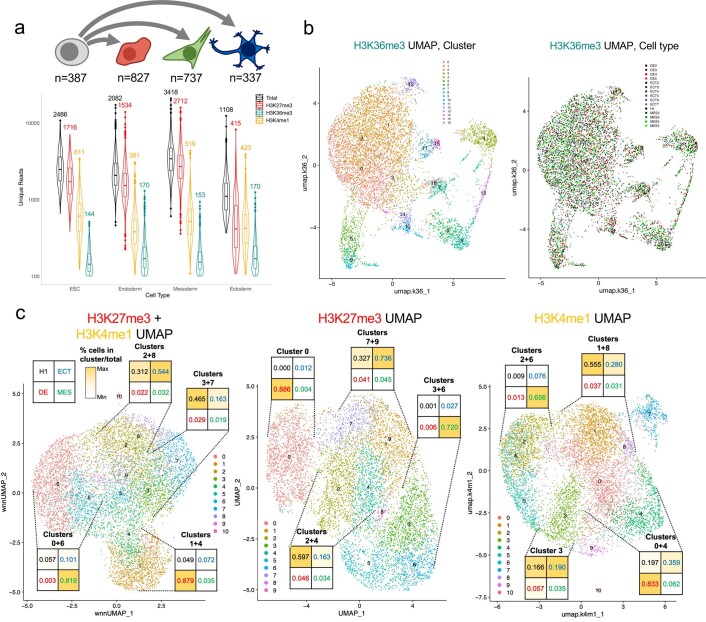
Clusters derived from scMulTI-Tag in hESC trilineage differentiation. a) Violin plots describing distributions of unique reads per cell in H1 hESCs (left), endoderm (center-left), mesoderm (center-right), or ectoderm (right) for all cells with at least 100 unique reads originating from each of the three targets used in the experiments described in Fig. 4. Median values for total unique reads (black), H3K27me3 unique reads (red), H3K4me1 unique reads (orange) or H3K36me3 unique reads (teal) are displayed at the top of each violin. Number of cells described is displayed at top of each cell type group. b) UMAP plot for single cell MulTI-Tag data from projection of H3K36me3 data, with cells colored by Seurat cluster (left) or cell type (right). c) UMAP plots for single cell MulTI-Tag data from projection of H3K27me3 data (center), H3K4me1 data (right), or a weighted nearest neighbor integration of H3K27me3 and H3K4me1 data (left). Cells are colored by Seurat clusters. For each plot, four groups of representative clusters are highlighted with quadrants describing the fraction of H1 (top left), ectoderm (top right), endoderm (bottom left), or mesoderm (bottom right) cells contained in the highlighted clusters as a proportion of the total cells from each cell type contained in the experiment. Quadrants are colored based on the proportion of the maximum value in the quadrant.

**Extended Data Fig. 8:**
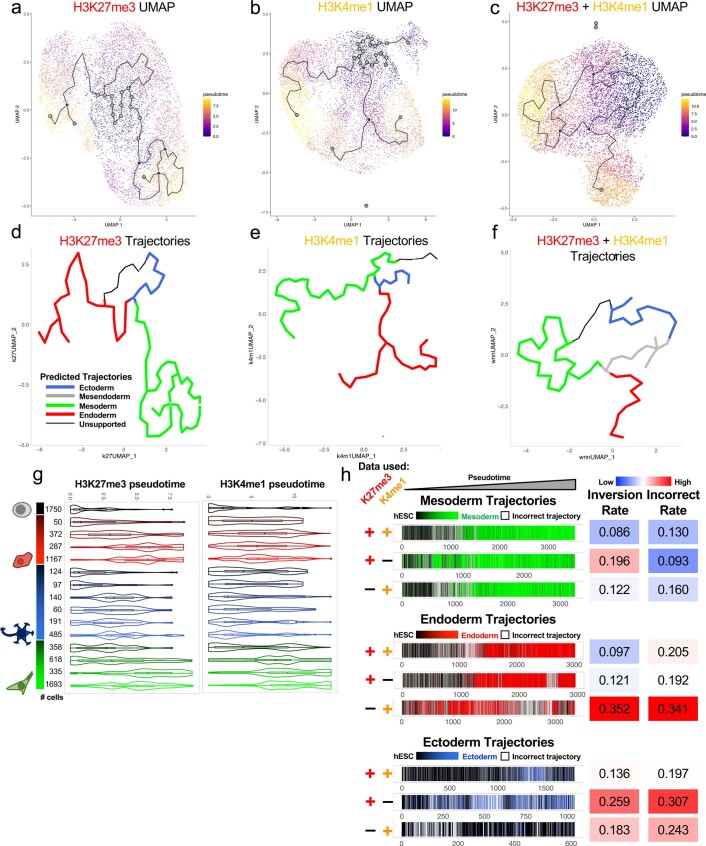
Pseudotemporal trajectories derived from scMulTI-Tag in hESC trilineage differentiation. a) UMAP plot for single cell MulTI-Tag data from projection of H3K27me3 data, with monocle3-derived pseudotemporal trajectories overlaid. Cells are colored by inferred pseudotime. b) UMAP plot describing the same as b) for H3K4me1 data. c) UMAP plot describing the same as a) and b) for a weighted nearest neighbor integration of H3K27me3 and H3K4me1 data. d) monocle3-derived pseudotemporal trajectories for H3K27me3 data, colored by manual annotation of likely correspondence to known differentiation trajectories. e) Same as d) for H3K4me1 data. f) same as d) and e) for a weighted nearest neighbor integration of H3K27me3 and H3K4me1 data. g) Violin plots showing the distribution of inferred pseudotimes derived from H3K27me3 (left) or H3K4me1 (right) data for each cell type profiled. Number of cells profiled for each cell type is denoted at left. h) Pseudotime-ordered heatmaps describing the cell types of the cells assigned to each manually curated trajectory derived from different MulTI-Tag data. Data used to derive each trajectory is displayed at left. For each trajectory, cells are colored by color intensity based on the real assayed differentiation time ranging from hESC (black) to the terminal cell type (mesoderm = green; endoderm = red; ectoderm = blue). Cells assigned to the inferred trajectory that belong to a different trajectory (‘incorrect’) are colored white. For each trajectory-data source combination, inversion rate, defined as the fraction of cell pairs in the trajectory for which the real differentiation time is out of order, and incorrect rate, defined as the fraction of cells assigned to an incorrect trajectory, are displayed at right.

To ascertain how histone modifications co-occur in single cells in a continuous developmental context, we differentiated H1 hESCs into three germ layers (Endoderm, Mesoderm and Ectoderm); harvested nuclei at 24-hour timepoints across the three time courses; and used MulTI-Tag to co-profile H3K27me3, H3K4me1 and H3K36me3, resulting in 7,727 cells meeting quality filters (Fig. 4a and Extended Data Fig. 7a). A UMAP based on H3K36me3 was unable to distinguish cell types as calculated by NMI for distinct cluster assignment of the four terminal cell types (NMI = 0.0166; Extended Data Fig. 7b). However, UMAPs based on H3K27me3 (NMI = 0.4060), H3K4me1 (NMI = 0.277) or WNN synthesis of H3K27me3 and H3K4me1 signal (NMI = 0.3403) all distinguished two major clusters corresponding to endoderm and mesoderm, along with H1-dominant or ectoderm-dominant clusters that were partially mixed, consistent with H1 hESC gene expression profiles being more similar to ectoderm^42^ (Fig. 4b and Extended Data Fig. 7b). To determine how well MulTI-Tag profiles reflect expected developmental trajectories, we used H3K27me3, H3K4me1 or combined H3K27me3–H3K4me1 MulTI-Tag data to infer pseudotemporally ordered differentiation trajectories using monocle3 (ref. ^43^). We then calculated two quality metrics: frequency of cell type assignment to an incorrect trajectory and inversion frequency, or the likelihood that ‘correct’ trajectory timepoints derived from known differentiation age were ‘out of order’ based on the inference (Fig. 4d and Extended Data Fig. 8a–f). Relative to either H3K27me3 or H3K4me1 pseudotime alone, inferred H3K27me3–H3K4me1 pseudotime correlated more closely with known differentiation age based on experimental timepoints (Fig. 4c and Extended Data Fig. 8g) and minimized both incorrect trajectory assignment and trajectory-specific inversion rates (Extended Data Fig. 8h). Moreover, the H3K27me3–H3K4me1 inferred trajectories alone recapitulated two major known branch points in hESC tri-lineage differentiation: partitioning of Ectoderm and Mesendoderm lineages at the outset of differentiation based on TGF-β and WNT signaling and subsequent separation of Endoderm and Mesoderm based on BMP and FGF signaling^44,45^ (Fig. 4d). These results show that multifactorial data integration is important for accurately representing continuous developmental chromatin states.

**Extended Data Fig. 9:**
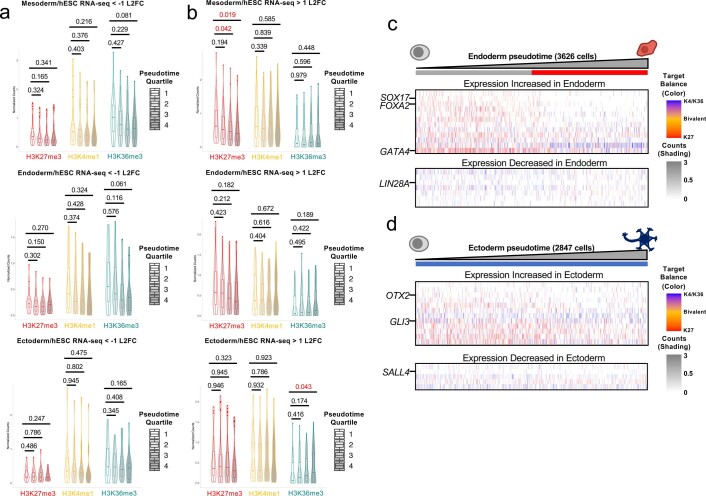
Analysis of scMulTI-Tag changes across pseudotime. a) Violin plots describing H3K27me3 (red), H3K4me1 (orange), and H3K36me3 (teal) single cell MulTI-Tag enrichment in genes that decline in expression as defined by RNA-seq^44^ during differentiation from hESCs to mesoderm (top, n = 29), endoderm (middle, n = 20), or ectoderm (bottom, n = 19). Enrichment is partitioned by pseudotime quartile (1=lowest, 4=highest). P-values of Wilcoxon Rank Sum test between quartile 1 and all other quartiles for each target are displayed above violins. b) Violin plots describing same as a) for genes that increase in expression as defined by RNA-seq^44^ . Mesoderm n = 54, Endoderm n = 35, Ectoderm n = 36. P-values less than 0.05 are highlighted in red. c) Heatmaps describing co-occurrence of MulTI-tag targets in selected genes of interest whose RNA-seq expression increases (top) or decreases (bottom) during differentiation from hESC to endoderm in 3626 single cells classified as hESC or different stages of differentiated mesoderm. Heatmaps are sorted left-to-right by increasing pseudotime in the mesendoderm/endoderm trajectory. The balance of enrichment between H3K4me1/H3K36me3 and H3K27me3 in each cell is denoted by color, and the total normalized counts in each cell are denoted by the transparency shading. d) Same as c) for ectoderm trajectory.

To determine how continuous transitions in chromatin enrichment across differentiation correlate with changes in developmental gene expression, we quantified changes in H3K27me3, H3K4me1 and H3K36me3 enrichment across pseudotime in transcription factors (TFs) with the highest reported fold change enrichment in RNA-seq^44^ between a terminal cell type (endoderm, mesoderm or ectoderm) and hESCs. Notably, there were trajectory-specific differences in enrichment changes: for TFs whose expression declines during differentiation as measured by RNA-seq, we observed a decline in H3K36me3 enrichment across pseudotime accompanied by relatively low and stable levels of H3K4me1 and H3K27me3 in the mesoderm and endoderm trajectories, whereas the ectoderm trajectory was characterized only by a decline in H3K4me1 enrichment (Extended Data Fig. 9a). For TFs whose expression increases, H3K27me3 is lost gradually in a pseudotime-dependent manner in endoderm and mesoderm trajectories, whereas, in the ectoderm trajectory, H3K27me3 is low at the onset of differentiation, and H3K36me3 enrichment increases across pseudotime (Extended Data Fig. 9b). These phenomena were particularly pronounced for core regulators of cell identity, including *LEF1* in mesoderm and *SOX17* and *FOXA2* in endoderm, whereas ectoderm regulators, such as *OTX2*, were largely devoid of H3K27me3 early in the ectoderm trajectory (Fig. 4e and Extended Data Fig. 9c,d), indicating that different trajectories manifest distinct temporal chromatin trends at genes important for differentiation.

**Extended Data Fig. 10:**
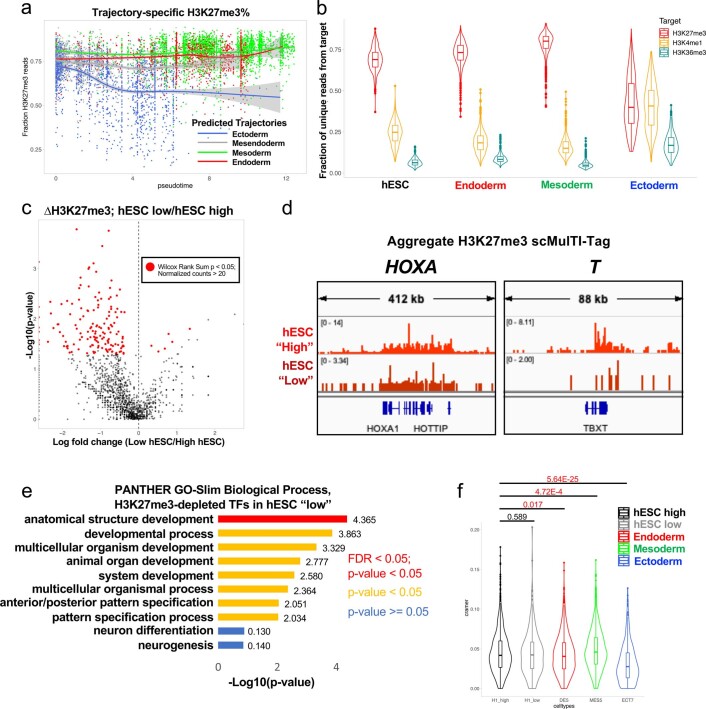
Trajectory-specific H3K27me3 dynamics uncovered by MulTI-Tag. a) Scatterplot showing single cells plotted by increasing pseudotime on the X-axis, increasing fraction of H3K27me3 as a proportion of total unique reads on the Y-axis, and colored by trajectory to which they belong (Ectoderm = blue, Mesendoderm = grey, Mesoderm.= green, Endoderm = red). LOESS smoothing curves describing average results for each trajectory are overlaid. b) Violin plots describing the distribution of the proportions of MulTI-Tag H3K27me3 (red), H3K4me1 (orange), or H3K36me3 (teal) unique reads out of total unique reads in individual H1 hESC (left, n = 1750) endoderm (center-left, n = 1167), mesoderm (center-right, n = 1693), or ectoderm (right, n = 485) cells. c) Volcano plot showing all human transcription factors plotted by log fold change in H3K27me3 MulTI-Tag normalized enrichment between ‘H3K27me3-low’ and ‘H3K27me3-high’ H1 hESCs on the X-axis, and negative log10 Wilcoxon Rank-Sum p-value of the comparisons on the y-axis. Genes for which the total normalized counts are greater than 20 and the p-value is less than 0.05 are highlighted in red. d) Genome browser shots showing aggregate H3K27me3 MulTI-Tag enrichment in ‘H3K27me3-high’ (red) and ‘H3K27me3-low’ (dark red) cells at the *HOXA* (left) and *TBXT* (*T*, right) loci. e) Gene Ontology analysis of transcription factors with a statistically significant reduction in H3K27me3 in ‘H3K27me3-low’ hESCs as compared to all human TFs, with p-values, length of bars and reported values at right of bars corresponding to negative Log10(p-value) for each category displayed. Bars are colored by FDR and p-value thresholds as denoted. f) Violin plots describing calculated Cramér’s V of association between H3K27m3 and H3K4me1 in individual H3K27me3-high hESCs (black), H3K27me3-low hESCs (grey), endoderm (red), mesoderm (green), and ectoderm cells. Wilcoxon Rank-Sum p-values of comparisons between ‘H3K27me3-high’ hESCs and other cell types are displayed at top. P-values less than 0.05 are highlighted in red.

The unique enrichment profile of the ectoderm trajectory led us to wonder whether changes in global histone modification enrichment may be similarly distinct. As with our experiments in H1 and K562 cells, we calculated the percentage of unique reads assigned to each of the three targets in single cells and analyzed how target balance changed across trajectories. We found that the ectoderm trajectory exhibited a rapid, pseudotime-dependent reduction in H3K27me3 as a percentage of all targets (Extended Data Fig. 10a), resulting in terminal ectoderm exhibiting significantly lower H3K27me3 percentage than other cell types (Fig. 4d and Extended Data Fig. 10b). Notably, hESCs predicted to participate in the ectoderm trajectory also had a lower percentage of H3K27me3 than those participating in the mesendoderm trajectory (*P* < 1 × 10^−5^, Wilcoxon rank-sum test) (Fig. 4f). To ascertain whether H3K27me3 level was correlated with developmental gene regulation, we partitioned hESCs into ‘low’ and ‘high’ H3K27me3 groupings, calculated normalized differences in gene-specific enrichment and examined a panel of known regulators of germ cell differentiation (Fig. 4g and Extended Data Fig. 10c). Curiously, whereas most genes exhibited a negligible or modest decline in enrichment despite different global H3K27me3 levels, including constitutively silenced genes such as *HOXB3*, TFs specifically active in the first phase of germ layer specification after pluripotency exit, including *TBXT (T)* and *OTX2*, were strongly de-repressed in the ‘low’ population of cells (Fig. 4f and Extended Data Fig. 10d), suggesting that low H3K27me3 in hESCs is accompanied by a uniquely configured developmental state. TFs de-repressed in the ‘low’ population were enriched for Gene Ontology terms related to organ/anatomical development and pattern specification but not for terms related to neurogenesis, suggesting that such cells were generally primed for differentiation rather than representing spuriously differentiated ectoderm (Extended Data Fig. 10e). Finally, we quantified intragenic ‘bivalent’ H3K27me3–H3K4me1 co-occurrence across cell types and found that ectoderm bivalency is significantly lower than hESCs, endoderm or mesoderm, consistent with the original observation that bivalency is absent in neuronally derived lineages^27^ (Extended Data Fig. 10f). Bivalency was equivalent in H3K27me3-low and H3K27me3-high hESC populations, however, indicating that pluripotency-specific chromatin characteristics are maintained in H3K27me3-low hESCs despite their distinct chromatin environment (Extended Data Fig. 10f). Taken together, these results show that global changes in chromatin modification enrichment and co-enrichment that can be detected before differentiation are associated with specific developmental endpoints.

## Discussion

MulTI-Tag establishes a rigorous baseline for unambiguously profiling multiple epigenome proteins with direct sequence tags, maintaining both exemplary assay efficiency and target-assignment fidelity relative to other similar approaches^21,46^. We use a well-documented combinatorial barcoding strategy^3,47^ that can be implemented without any specialized equipment by substituting standard polymerase chain reaction (PCR) plates for the ICELL8 apparatus. Three targets profiled here—H3K27me3, H3K4me1/2 and H3K36me3—are typically enriched at distinct stages of the gene regulatory cycle that proceeds from developmental repression (H3K27me3) to enhancer and promoter activation (H3K4me1/2) to productive transcription elongation (H3K36me3). We integrated this temporal information across a model of ESC differentiation to germ layers to characterize continuous changes in chromatin enrichment that corresponded with specific differentiation outcomes, including a global low-H3K27me3 signature in hESCs associated with ectoderm differentiation. This is perhaps consistent with a ‘goldilocks’ zone that balances an immediate need to prevent spurious mesendoderm signaling^48^ with a need to mitigate silencing later during neurogenesis^49^. By simultaneously measuring locus-specific enrichment and the relative abundances of multiple targets, multifactorial profiling is uniquely suited to characterize this style of context-specificity in developmental chromatin regulatory strategies. Whereas pseudotemporal inference using MulTI-Tag was sufficient to build accurate trajectories, we suspect that molecular ‘velocity’ analyses may be more challenging to implement if the context-specificity that we observe violates steady-state assumptions on which they are based^50,51^. Finally, our analysis of co-occurrence of different targets in the same genes elucidates chromatin enrichment at single-locus, single-cell resolution and further allowed us to confirm classic ‘bivalent’ co-enrichment and detect an unexpected class of H3K27me3–H3K36me3 co-enriched genes that we verified via public ENCODE data. H3K27me3–H3K36me3 are considered to be antagonistic within the same histone tail^40,52^, and, because we found here that their co-enrichment occurs on different nucleosomes in the same gene, it is unclear whether this is a bona fide ‘bivalent’ state or, rather, a dynamic intermediate state. Nevertheless, our findings are consistent with previously reported H3K27me3 spreading via Tudor domain-containing subunits of the polycomb repressive complex (PRC) engaging H3K36me3 in ESCs^53–55^. We anticipate further work to understand intra-locus interactions between different chromatin characteristics to bear on longstanding hypotheses regarding bivalency^27^ and hyperdynamic chromatin^56^.

Opportunities for refinement of MulTI-Tag exist. Although MulTI-Tag is theoretically scalable to any combination of user-defined targets in the same assay, in practice, downstream analysis is constrained by the decreasing number of cells that meet minimum read criteria for every target. Therefore, one should expect higher ‘computational loss’ of cells when profiling more than three targets as presented here and adjust cellular input accordingly. It is possible that methods to mitigate target-specific ‘jackpotting’ amplification bias^57^ could resolve this. Our emphasis on ensuring both that the efficiency of MulTI-Tag profiling was similar to CUT&Tag and that there was minimal cross-contamination between antibody-assigned adapters led us to generate antibody–adapter conjugates^46^ and to incubate and tagment with antibody–adapter–transposase complexes sequentially rather than simultaneously. By physically excluding the possibility of adapter or Tn5 monomer exchange in the protocol, MulTI-Tag safeguards against potential artifacts originating from adapter crossover, identifying any set of user-defined targets with high fidelity. However, alternative reagent schemes that allow simultaneous antibody incubations and tagmentation while maintaining target fidelity may increase the number of targets that can be profiled in a single experiment. Innovations in protein engineering, such as fusing Tn5 directly to an antibody, may aid such efforts^58,59^. In the future, we anticipate that development of chromatin-integrated multimodal^30,60^ and spatial^61^ single-cell technologies will benefit substantially from multifactorial profiling by pairing its potential benefits in cross-factor developmental analysis with strong existing cell type identification and tissue-contextual molecular signatures.

## Conclusions

MulTI-Tag is an effective tool for refining understanding of chromatin regulation at single-cell, single-locus resolution.

## Methods

### Cell culture and nuclei preparation

Human female K562 chronic myleogenous leukemia cells (American Type Culture Collection (ATCC)) were authenticated for STR, sterility, human pathogenic virus testing, mycoplasma contamination and viability at thaw. H1 (WA01) male hESCs (WiCell) were authenticated for karyotype, STR, sterility, mycoplasma contamination and viability at thaw. K562 cells were cultured in liquid suspension in IMDM (ATCC) with 10% FBS added (Seradigm). H1 cells were cultured in Matrigel (Corning)-coated plates at 37 °C and 5% CO_2_ using mTeSR-1 Basal Medium (STEMCELL Technologies) exchanged every 24 hours. K562 cells were harvested by centrifugation for 3 minutes at 1,000*g* and then resuspended in 1× PBS. H1 cells were harvested with ReleasR (STEMCELL Technologies) using the manufacturer’s protocols. H1 cells were differentiated to germ layers using the STEMDiff Trilineage Differentiation Kit (STEMCELL Technologies) according to the manufacturer’s protocols. Lightly cross-linked nuclei were prepared from cells as described in steps 2–14 of the Bench Top CUT&Tag protocol on protocols.io (10.17504/protocols.io.bcuhiwt6). In brief, cells were pelleted for 3 minutes at 600*g*, resuspended in hypotonic NE1 buffer (20 mM HEPES-KOH pH 7.9, 10 mM KCl, 0.5 mM spermidine, 10% Triton X-100 and 20% glycerol) and incubated on ice for 10 minutes. The mixture was pelleted for 4 minutes at 1,300g, resuspended in 1× PBS and fixed with 0.1% formaldehyde for 2 minutes before quenching with 60 mM glycine. Nuclei were counted using the ViCell Automated Cell Counter (Beckman Coulter) and frozen at −80 °C in 10% DMSO for future use.

### Antibodies

Antibodies used for CUT&Tag or MulTI-Tag in this study were as follows: rabbit anti-H3K27me3 (Cell Signaling Technologies, CST9733S, lot 16, 1:100 dilution), mouse anti-RNA PolIIS5P (Abcam, ab5408, lot GR3264297-2, 1:100 dilution), mouse anti-H3K4me2 (Active Motif, 39679, lot 31718013, 1:100 dilution), mouse anti-H3K36me3 (Active Motif, 61021, lot 23819012, 1:100 dilution), rabbit anti-H3K9me3 (Abcam, ab8898, lot GR3302452-1, 1:100 dilution), rabbit anti-H3K4me1 (EpiCypher, 13-0040, lot 2134006-02, 1:100 dilution), guinea pig anti-rabbit (Antibodies Online, ABIN101961, 1:100 dilution) and rabbit anti-mouse (Abcam, ab46450, 1:100 dilution). For antibody–adapter conjugation, antibodies were ordered from manufacturers with the following specifications if not already available as such commercially: 1× PBS, no BSA, no sodium azide and no glycerol. For secondary conjugate MulTI-Tag, secondary antibody conjugates from the TAM-ChIP rabbit and mouse kits (Active Motif) were used.

### CUT&Tag

CUT&Tag was carried out as previously described^17^ (10.17504/protocols.io.bcuhiwt6). In brief, nuclei were thawed and bound to washed paramagnetic concanavalin A (ConA) beads (Bangs Laboratories) and then incubated with primary antibody at 4 °C overnight in Wash Buffer (10 mM HEPES pH 7.5, 150 mM NaCl, 0.5 mM spermidine and Roche Complete Protease Inhibitor Cocktail) with 2 mM EDTA. Bound nuclei were washed and incubated with secondary antibody for 1 hour at room temperature and then washed and incubated in Wash-300 Buffer (Wash Buffer with 300 mM NaCl) with 1:200 loaded pA–Tn5 for 1 hour at room temperature. Nuclei were washed and tagmented in Wash-300 Buffer with 10 mM MgCl_2_ for 1 hour at 37 °C and then resuspended sequentially in 50 µl of 10 mM TAPS and 5 µl of 10 mM TAPS with 0.1% SDS and incubated for 1 hour at 58 °C. The resulting suspension was mixed well with 16 µl of 0.9375% Triton X-100, and then primers and 2× NEBNext Master Mix (New England Biolabs) were added for direct amplification with the following conditions: (1) 58 °C for 5 minutes, (2) 72 °C for 5 minutes, (3) 98 °C for 30 seconds, (4) 98 °C 10 seconds, (5) 60 °C for 10 seconds, (6) repeat steps 4–5 14 times, (7) 72 °C for 2 minutes and (8) hold at 8 °C. DNA from amplified product was purified using 1.1× ratio of HighPrep PCR Cleanup System (MagBio) and resuspended in 25 µl of 10 mM Tris-HCl with 1 mM EDTA, and concentration was quantified using the TapeStation system (Agilent). For sequential and combined CUT&Tag, rather than incubating the secondary antibody and pA–Tn5 separately, pA–Tn5 was pre-incubated with an equimolar amount of secondary antibody in 50 µl of Wash-300 buffer at 4 °C overnight. For sequential, primary antibody incubation, secondary antibody pA–Tn5 incubation and tagmentation were carried out sequentially for each primary–secondary-barcoded pA–Tn5 combination, whereas, for combined, all reagents were incubated simultaneously for their respective protocol steps (that is, primary antibodies together and secondary antibody pA–Tn5 complexes together), and tagmentation was carried out once for all targets.

### Conjugates for MulTI-Tag

Antibody–adapter conjugates were generated by random amino-conjugation between 100 µg of antibody purified in PBS in the absence of glycerol, BSA and sodium azide and 5′ aminated, barcode-containing oligonucleotides (Integrated DNA Technologies) using the Oligonucleotide Conjugation Kit (Abcam) according to the manufacturer’s protocols. Before conjugation, 200 µM adapter oligos resuspended in 1× PBS were annealed to an equimolar amount of 200 µM Tn5MErev (5′-[phos]CTGTCTCTTATACACATCT-3′) in 1× PBS to yield 100 µM annealed adapters. In all cases, primary antibodies were conjugated with an estimated 10:1 molar excess of adapter to conjugate. The sequences of adapters used are listed in Supplementary Table 1.

### Bulk MulTI-Tag protocol

For each target to be profiled in MulTI-Tag, an antibody–i5 adapter conjugate was generated as described above, and 0.5 µg of conjugate was incubated with 1 µl of ~5 µM pA–Tn5 and 16 pmol unconjugated, Tn5MErev-annealed i5 adapter of the same sequence in minimal volume for 30 minutes to 1 hour at room temperature to generate conjugate-containing i5 transposomes. In parallel, a separate aliquot of 1 µl of pA–Tn5 was incubated with 32 pmol i7 adapter for 30 minutes to 1 hour at room temperature to generate an i7 transposome. Conjugate i5 and i7 transposomes were used in MulTI-Tag experiments within 24 hours of assembly. After transposome assembly, 50,000 nuclei were thawed and bound to washed ConA beads and then incubated with the first conjugate transposome resuspended in 50 µl of Wash-300 Buffer plus 2 mM EDTA for 1 hour at room temperature or overnight at 4 °C. After incubation, the nuclei mix was washed three times with 200 µl of Wash-300 Buffer and then tagmented in 50 µl of Wash-300 Buffer with 10 mM MgCl_2_ for 1 hour at 37 °C. After tagmentation, buffer was removed and replaced with 200 µl of Wash-300 with 5 mM EDTA and incubated for 5 minutes with rotation. The conjugate incubation and tagmentation protocol was then repeated for the remainder of conjugates to be used, up to the point of incubation with the final conjugate. The optimal order of conjugate tagmentation was ascertained empirically by observing the optimal balance of reads between targets and, in this study, were tagmented in the following order: PolIIS5P–H3K27me3; H3K9me3–H3K27me3; H3K4me1–H3K27me3; H3K36me3–H3K27me3; H3K4me2–H3K36me3-H3K27me3; or H3K4me1–H3K36me3–H3K27me3. After incubation, the supernatant was cleared, and secondary antibodies corresponding to the species in which the primary antibody conjugates were raised were added in 100 µl of Wash Buffer and incubated for 1 hour at room temperature. The nuclei were then washed twice with 200 µl of Wash Buffer, and the i7 transposome was added in 100 µl of Wash-300 Buffer and incubated for 1 hour at room temperature. After three washes with 200 µl of Wash-300 Buffer, the final tagmentation is carried out by adding 50 µl of Wash-300 Buffer with 10 mM MgCl_2_ and incubating for 1 hour at 37 °C. After tagmentation, the nuclei are resuspended in 10 mM TAPS, denatured in TAPS-SDS, neutralized in Triton X-100 and amplified, and libraries are purified as described above. All nuclei transfers were carried out in LoBind 0.6-ml tubes (Axygen). For combined MulTI-Tag, all antibody conjugate incubation and tagmentation steps were carried out simultaneously.

### Single-cell MulTI-Tag

Single-cell MulTI-Tag was carried out as described in the bulk MulTI-Tag protocol up to the completion of the final tagmentation step, with the following modifications: 250 µl of paramagnetic streptavidin T1 Dynabeads (Sigma-Aldrich) was washed three times with 1 ml of 1× PBS and resuspended in 1 ml of 1× PBS with 0.01% Tween 20; 240 µl of biotin-wheat germ agglutinin (WGA) (Vector Labs) combined with 260 µl of 1× PBS was incubated with Dynabeads for 30 minutes and resuspended in 1 ml of 1× PBS with 0.01% Tween 20 to generate WGA beads; and 100 µl of washed beads was pre-bound with 1.8 million nuclei. For each experiment, 15 µg of H3K4me2 and H3K36me3 conjugate and 7.5 µg of H3K27me3 conjugate were used and loaded into transposomes at the ratios described above. All incubations were carried out in 200 µl and washes in 400 µl. After final conjugate and secondary antibody incubation, nuclei were distributed equally across i7 transposomes containing 96 uniquely barcoded adapters (Supplementary Table 1). After the final tagmentation step, nuclei were re-aggregated into a single tube, washed twice in 100 µl of 10 mM TAPS and transferred to a cold block chilled to 0 °C on ice. Supernatant was removed, and nuclei were incubated in ice-cold DNase reaction mix (10 µl of RQ1 DNase (Promega), 10 µl of 10× DNase buffer and 80 µl of ddH_2_O) for 10 minutes in a cold block. The reaction was stopped by adding 100 µl of ice-cold RQ1 DNase Stop Buffer. Nuclei were immediately washed once in 100 µl of 10 mM TAPS and then resuspended in 650 µl of TAPS. Two 20-µm cell strainers (Thermo Fisher Scientific) were affixed to fresh 1.5-mL LoBind tubes, and 325 µl of nuclei mix was added to the top of each. Tubes were spun for 10 minutes at 300*g* to force nuclei through the strainers and then the flowthrough was combined and resuspended in 640 µl of 10 mM TAPS. To the final nuclei mix, 16 µl of 100× DAPI and 8 µl of ICELL8 Second Diluent (Takara) were added and incubated for 10 minutes at room temperature. Nuclei were quantified on a Countess 3 cell counter (Thermo Fisher Scientific), and the nuclei mix was adjusted to a concentration of 857 nuclei per microliter. Then, 640 µl of nuclei were dispensed into an ICELL8 microfluidic chip according to the manufacturer’s protocols, and SDS denaturation, Triton X-100 neutralization and amplification were carried out in microwells as described previously^62^. After amplification, microwell contents were re-aggregated, and libraries were purified with two rounds of cleanup with 1.3× HighPrep beads and resuspended in 20 µl of 10 mM Tris-HCl with 1 mM EDTA.

### Sequencing and data pre-processing

Libraries were sequenced on an Illumina HiSeq instrument with paired-end 25 × 25 reads. Sequencing data were aligned to the UCSC hg19 genome build using Bowtie2 (ref. ^63^), version 2.2.5, with parameters –end-to-end–very-sensitive–no-mixed–no-discordant -q–phred33 -I 10 -X 700. Mapped reads were converted to paired-end BED files containing coordinates for the termini of each read pair and then converted to bedGraph files using BEDTools genomecov with parameter –bg^64^. For single-cell experiments, mapped reads were converted to paired-end Cell Ranger-style BED files, in which the fourth column denotes cell barcode combination, and the fifth column denotes the number of fragment duplicates. Raw read counts and alignment rates for all sequencing datasets presented in this study are listed in Supplementary Table 2.

### Data analysis

Single-cell MulTI-Tag pre-processing, feature selection, dimensionality reduction and UMAP projection were carried out as follows. For each target, we selected a cutoff of 100 unique fragments per cell, and cells were retained only if they met unique read count criteria for all three targets, with the exception of the germ layer differentiation experiments in which the unique read cutoff for H3K36me3 was relaxed to maximize the number of cells analyzed for dimensionality reduction and trajectory analysis. For bulk MulTI-Tag, peaks were called using SEACR version 1.4 (ref. ^65^) with the following settings: -n norm, -m stringent, -e 0.1 (https://github.com/FredHutch/SEACR). For single-cell MulTI-Tag, peaks were called from aggregate profiles from unique read count-filtered cells using SEACR version 1.4 with the following settings: -n norm, -m stringent, -e 5. Peak calls presented in this study are listed in Supplementary Table 3. All dimensionality reduction, UMAP analysis and clustering was performed using Seurat version 4.0.5 and Signac version 1.5.0, with the exception of datasets described in Extended Data Fig. 4. Those datasets were analyzed as follows. Cell-specific unique reads were intersected with a BED file representing 50-kb windows spanning the hg19 genome using BEDTools^64^ to generate BED files in which each line contained a unique window-cell-read count instance. In R (https://www.r-project.org), these BED files were cast into peak (rows) by cell (columns) matrices (using the reshape library version 3.6.2), which were filtered for the top 40% of windows by aggregate read counts, scaled by term frequency-inverse document frequency (TF-IDF) and log-transformed. Transformed matrices were subjected to singular value decomposition (SVD), and SVD dimensions for which the values in the diagonal matrix ($d as output from the ‘svd’ command in R) were greater than 0.2% of the sum of all diagonal values were used as input to the ‘umap’ command from the UMAP library in R. For clustering analyses of K562-H1 datasets, we used k-means clustering to define two clusters for each dataset and then calculated NMI using the ‘NMI’ function from the ‘aricode’ library in R, based on the cluster and real cell type classifications for each cell. For the germ layer differentiation experiment, we used Seurat-derived cluster annotations and considered only cells classified as hESC, Endoderm, Ectoderm or Mesoderm. For genic co-occurrence analysis, fragments were mapped to genes in a window extending from 1 kb upstream of the farthest distal annotated TSS to the annotated transcription end site (TES). The statistical significance of cell-specific, target-specific fragment accumulation in genes was verified by calculating the probability of *X* fragment–gene overlaps in cell *I* based on a Poisson distribution with a mean *µ*_*i*_ defined by the cell-specific likelihood of a fragment overlap with any base pair in the hg19 reference genome:

$$p = Poisson\left( {X \ge x,\mu _i} \right);\,{{{\mathrm{where}}}}\,x = \frac{{r \ast L_i}}{{L_{gene}}}\,{{{\mathrm{and}}}}\,\mu _i = \frac{{L_i \ast f_i}}{{L_{genome}}}$$

where *L*_*i*_ = median fragment size in cell *i*; *f*_*i*_ = number of fragments mapping in cell *i*; *L*_*gene*_ = length of the gene being tested; and *L*_*genome*_ = length of the reference genome. All gene–fragment overlaps considered in this study were determined to be statistically significant at a *P* < 0.01 cutoff after Benjamini–Hochberg multiple testing correction. *P* values comparing fraction of reads in peaks in Extended Data Fig. 1f, target combination proportions in single cells in Extended Data Fig. 5, normalized count enrichment in Extended Data Fig. 6c, normalized count enrichment in Extended Data Fig. 9a,b and Cramér’s V in Extended Data Fig. 10f were calculated using two-sided *t*-tests. All *P* values from two-sided *t*-tests were determined without multiple testing correction. All underlying statistics associated with statistical comparisons presented in this study are listed in Supplementary Table 4. Genome browser screenshots were obtained from Integrative Genomics Viewer (IGV)^66^. CUT&Tag/MulTI-Tag enrichment heat maps and average plots were generated in DeepTools^67^. UMAPs, violin plots, box plots and scatter plots were generated using ggplot2 (https://ggplot2.tidyverse.org). For all box plots, the center line reflects the data mean; the upper and lower bounds of the box represent the 0.75 and 0.25 quantiles of the data, respectively; and the whisker minima and maxima reflect 1.5× the interquartile range (the 0.75 quantile minus the 0.25 quantile) below the 0.25 quantile or above the 0.75 quantile, respectively.

### Reporting summary

Further information on research design is available in the Nature Research Reporting Summary linked to this article.

## Online content

Any methods, additional references, Nature Research reporting summaries, source data, extended data, supplementary information, acknowledgements, peer review information; details of author contributions and competing interests; and statements of data and code availability are available at 10.1038/s41587-022-01522-9.

## Supplementary information

Reporting Summary

Supplementary Table 1 Sequences for pA–Tn5 adapters and amplification primers used in this study. P5_i5 and P7_i7 adapters are annealed to the Tn5MErev oligo and loaded into pA–Tn5, whereas i5 and i7 primers are used for amplification in bulk or single-cell MulTI-Tag protocols. P5_i5 adapters were used in association with specific targets as denoted. P7_i7 adapters were used for either bulk or single-cell MulTI-Tag as denoted. Primers were used in specific combinations for bulk MulTI-Tag, and all 72 i5 and 72 i7 primers were dispensed on an ICELL8 chip according to the manufacturer’s instructions for single-cell MulTI-Tag.

Supplementary Table 2 Information for sequence files used in this study. Fields reported are as follows: (1) File name prefix: Sequence file name as found on the Gene Expression Omnibus; (2) Sample name: Brief descriptive name; (3) Cell type: Cell type used in experiment; (4) Cell #: Number of cells used in experiment; (5) Target: Antigen for antibody used in experiment; (6) Manufacturer: Commercial manufacturer of antibody used in experiment; (7) Cat #: Antibody catalog number; (8) Lot #: Lot number for antibody used; (9) Amount used: Amount of antibody (in µg) used in experiment; (10) Time tagment: Duration of tagmentation incubation step; (11) Protocol: Main experimental protocol used as listed in Methods; (12) Replicate: Biological replicate number; (13) Sequenced reads: Raw reads sequenced for experiment; (14) Reads mapped: Number of reads mapped to assigned reference genome; and (15) Alignment rate: Percentage of raw reads properly mapped to reference genome.

Supplementary Table 3 Peak calls and associated statistics for experiments presented in this manuscript. All peak calling was conducted using SEACR version 1.4 (https://github.com/FredHutch/SEACR).

Supplementary Table 4 Quantile values, standard deviations, *P* values, test statistics, degrees of freedom and confidence intervals associated with all distributional data presented in this manuscript. Data quantiles presented here are 0.05, 0.25, 0.5 (mean), 0.75 and 0.95.

## Data Availability

All primary sequence data and interpreted track files for sequence data generated in this study have been deposited at the Gene Expression Omnibus: GSE179756 (ref. ^68^). Publicly available CUT&Tag data analyzed in this study are available at GSE124557. Publicly available ChIP-seq data analyzed in this study can be found at the ENCODE portal^69^ under the following accession numbers: K562 H3K27me3: ENCFF322IFF; K562 H3K36me3: ENCFF498CMP; K562 H3K4me2: ENCFF099LMD; K562 PolIIS5P: ENCFF542DOG; H1 H3K27me3: ENCFF559PMU; H1 H3K36me3: ENCFF804GLR; and H1 H3K4me2: ENCFF433NOA.

## Code availability

All interpreted data and code critical to the replication of the study are publicly available in a Zenodo repository^70^ (10.5281/zenodo.6636675).
