# Supplementary material for: Multifactorial profiling of epigenetic landscapes at single-cell resolution using MulTI-Tag
Source: Nat Biotechnol. 2022 Oct 31;41(5):708–16. doi: 10.1038/s41587-022-01522-9 (PMC10188359; doi:10.1038/s41587-022-01522-9)
Supplement: Supplementary file 1 — Reporting Summary [file 41587_2022_1522_MOESM1_ESM.pdf]

nature portfolio

Corresponding author(s): Henikoff, Steven

Last updated by author(s): Sep 19, 2022

Reporting Summary

Nature Portfolio wishes to improve the reproducibility of the work that we publish. This form provides structure for consistency and transparency in reporting. For further information on Nature Portfolio policies, see our Editorial Policies and the Editorial Policy Checklist.

Statistics

For all statistical analyses, confirm that the following items are present in the figure legend, table legend, main text, or Methods section.

n/a	Confirmed	
☐	☒	The exact sample size (*n*) for each experimental group/condition, given as a discrete number and unit of measurement
☒	☐	A statement on whether measurements were taken from distinct samples or whether the same sample was measured repeatedly
☐	☒	The statistical test(s) used AND whether they are one- or two-sided<br>*Only common tests should be described solely by name; describe more complex techniques in the Methods section.*
☒	☐	A description of all covariates tested
☐	☒	A description of any assumptions or corrections, such as tests of normality and adjustment for multiple comparisons
☐	☒	A full description of the statistical parameters including central tendency (e.g. means) or other basic estimates (e.g. regression coefficient) AND variation (e.g. standard deviation) or associated estimates of uncertainty (e.g. confidence intervals)
☐	☒	For null hypothesis testing, the test statistic (e.g. *F*, *t*, *r*) with confidence intervals, effect sizes, degrees of freedom and *P* value noted<br>*Give P values as exact values whenever suitable.*
☒	☐	For Bayesian analysis, information on the choice of priors and Markov chain Monte Carlo settings
☒	☐	For hierarchical and complex designs, identification of the appropriate level for tests and full reporting of outcomes
☐	☒	Estimates of effect sizes (e.g. Cohen's *d*, Pearson's *r*), indicating how they were calculated

Our web collection on statistics for biologists contains articles on many of the points above.

Software and code

Policy information about availability of computer code

Data collection	The size distributions and molar concentration of CUT&Tag/MulTI-Tag sequencing libraries were determined using an Agilent 4200 TapeStation. Up to 96 barcoded CUT&Tag/MulTI-Tag libraries were pooled at approximately equimolar concentration for sequencing. Paired-end 25×25 bp sequencing on the Illumina HiSeq 2500 platform was performed by the Fred Hutchinson Cancer Research Center Genomics Shared Resources. Single-cell MulTI-Tag libararies were prepared using unique i5 and i7 primer barcodes and pooled with bulk samples for sequencing.
Data analysis	Software packages used for analysis: bowtie2 2.4.2, bedtools 2.28.0, DeepTools 3.3.0, bedGraphToBigWig, Integrative Genomics Viewer 2.8.2, SEACR 1.4, R version 4.1.0 (R libraries used: reshape 0.8.8, ggplot2 3.3.6, umap 0.2.8.0, seurat 4.0.5, signac 1.5.0, monocle3 0.2.2, SeuratWrappers 0.3.0, EnsDb.Hsapiens.v75 2.99.0, BSgenome.Hsapiens.UCSC.hg19 1.4.3, Matrix 1.3.3, future 1.25.0, viridis 3.3.6)

For manuscripts utilizing custom algorithms or software that are central to the research but not yet described in published literature, software must be made available to editors and reviewers. We strongly encourage code deposition in a community repository (e.g. GitHub). See the Nature Portfolio guidelines for submitting code & software for further information.

Data

Policy information about availability of data

All manuscripts must include a data availability statement. This statement should provide the following information, where applicable:

- Accession codes, unique identifiers, or web links for publicly available datasets
- A description of any restrictions on data availability
- For clinical datasets or third party data, please ensure that the statement adheres to our policy

All primary sequence data and interpreted track files for sequence data generated in this study have been deposited at the Gene Expression Omnibus (GEO):

GSE179756. Publicly available CUT&Tag data analyzed in this study are found at GSE124557. Publicly available ChIP-seq data analyzed in this study are found at the ENCODE portal under the following accession numbers: K562 H3K27me3: ENCFF322IFF; K562 H3K36me3: ENCFF498CMP; K562 H3K4me2: ENCFF099LMD; K562 PolIIS5P: ENCFF542DOG; H1 H3K27me3: ENCFF559PMU; H1 H3K36me3: ENCFF804GLR; H1 H3K4me2: ENCFF433NOA. All interpreted data and code critical to the replication of the study are publicly available in a Zenodo Repository (doi.org://10.5281/zenodo.6636675).

Field-specific reporting

Please select the one below that is the best fit for your research. If you are not sure, read the appropriate sections before making your selection.

☒ Life sciences ☐ Behavioural & social sciences ☐ Ecological, evolutionary & environmental sciences

For a reference copy of the document with all sections, see nature.com/documents/nr-reporting-summary-flat.pdf

Life sciences study design

All studies must disclose on these points even when the disclosure is negative.

Sample size	No sample size calculation was performed. Samples were composed of single cell suspensions subject to loss during the protocol, and therefore exact sample size control was out of reach, and experiments proceeded based on the cells that were recovered.
Data exclusions	No data were excluded.
Replication	Where indicated in publicly available data, experiments were conducted in replicates. MulTI-Tag validation experiments were replicated 4 times in order to generate sufficient statistical power to compare with existing ChIP-seq and CUT&Tag datasets, and K562-H1 single cell MulTI-Tag experiments were replicated multiple times with different targets as described. All attempts at replication were successful.
Randomization	n/a. The data and analysis for this study is objective and not prone to influence by researcher bias.
Blinding	n/a. The data and analysis for this study is objective and not prone to influence by researcher bias.

Reporting for specific materials, systems and methods

We require information from authors about some types of materials, experimental systems and methods used in many studies. Here, indicate whether each material, system or method listed is relevant to your study. If you are not sure if a list item applies to your research, read the appropriate section before selecting a response.

Materials & experimental systems

n/a	Involved in the study	
☐	☒	Antibodies
☐	☒	Eukaryotic cell lines
☒	☐	Palaeontology and archaeology
☒	☐	Animals and other organisms
☒	☐	Human research participants
☒	☐	Clinical data
☒	☐	Dual use research of concern

Methods

n/a	Involved in the study	
☐	☒	ChIP-seq
☒	☐	Flow cytometry
☒	☐	MRI-based neuroimaging

Antibodies

Antibodies used	Rabbit anti-H3K27me3 (Cell Signaling Technologies CST 9733, Lot 16), Mouse anti-RNA PolIIS5P (Abcam ab5408, Lot GR3264297-2), Mouse anti-H3K4me2 (Active Motif 39679, Lot 31718013), Mouse anti-H3K36me3 (Active Motif 61021, Lot 23819012), Rabbit anti-H3K9me3 (Abcam ab8898, Lot GR3302452-1), Rabbit anti-H3K4me1 (EpiCypher 13-0040, Lot 2134006-02), Guinea Pig anti-Rabbit (Antibodies Online ABIN101961), Rabbit anti-Mouse (Abcam ab46450).
Validation	All antibodies are commercially available, and have been verified by Western blotting or by peptide ELISA described on the manufacturer's specification sheets. All antibodies used in this study are confirmed to recognize the human protein as stated on the manufactures website.

Eukaryotic cell lines

Policy information about cell lines

Cell line source(s)	K562 (ATCC), WA01 H1 hESC (WiCell)
Authentication	Human female K562 Chronic Myelogenous Leukemia cells (ATCC) were authenticared for STR, sterility, human pathogenic

Authentication	virus testing, mycoplasma contamination, and viability at thaw. H1 (WA01) male human embryonic stem cells (hESCs) (WiCell) were authenticated for karyotype, STR, sterility, mycoplasma contamination, and viability at thaw.
Mycoplasma contamination	Cell lines tested negative for mycoplasma contamination.
Commonly misidentified lines (See ICLAC register)	No commonly misidentified lines were used in this study.

ChIP-seq

Data deposition

☒ Confirm that both raw and final processed data have been deposited in a public database such as GEO.

☒ Confirm that you have deposited or provided access to graph files (e.g. BED files) for the called peaks.

Data access links
May remain private before publication.

All sequencing datasets have been submitted to the Gene Expression Omnibus (GEO) repository, accession number GSE179756 (https://urldefense.proofpoint.com/v2/url?u=https-3A__www.ncbi.nlm.nih.gov_geo_query_acc.cgi-3Facc-3DGSE179756&d=DwIBAg&c=eRAMFD45gAfqt84VtBcfhQ&r=_qTaZfV1zzGoUh7qwPBvOQ&m=kEISCdnInT5-I7gl6zSkhobwTA0rGUR9Q0zWgMdyOA8&s=3C4J7D4iHvzufF31MD8eRSTPjedUmM_l10vNteFgezs&e=), secure token ohynwswcvjcfnyp.
Code and processed data have been submitted to Zenodo, doi.org://10.5281/zenodo.6636675

Files in database submission

MM_Hs_MPM1069; K562_K27_CUTnTag_indiv; CUT&Tag for H3K27me3 in K562 cells, individually profiled
MM_Hs_MPM1070; K562_Ser5_CUTnTag_indiv; CUT&Tag for PolIIS5P in K562 cells, individually profiled
MM_Hs_MPM1071; K562_K27_MulTI_secondary_indiv; Secondary antibody conjugate MulTI-Tag for H3K27me3 in K562 cells, individually profiled
MM_Hs_MPM1072; K562_Ser5_MulTI_secondary_indiv; Secondary antibody conjugate MulTI-Tag for PolIIS5P in K562 cells, individually profiled
MM_Hs_MPM1073K27; K562_K27_CUTnTag_seq; H3K27me3-barcoded data from CUT&Tag for H3K27me3 and PolIIS5P in K562 cells, sequentially profiled
MM_Hs_MPM1073Ser5; K562_Ser5_CUTnTag_seq; PolIIS5P-barcoded data from CUT&Tag for H3K27me3 and PolIIS5P in K562 cells, sequentially profiled
MM_Hs_MPM1074K27; K562_K27_CUTnTag_comb; H3K27me3-barcoded data from CUT&Tag for H3K27me3 and PolIIS5P in K562 cells, simultaneously profiled
MM_Hs_MPM1074Ser5; K562_Ser5_CUTnTag_comb; PolIIS5P-barcoded data from CUT&Tag for H3K27me3 and PolIIS5P in K562 cells, simultaneously profiled
MM_Hs_MPM1075K27; K562_K27_MulTI_secondary_seq; H3K27me3-barcoded data from secconday antibody conjugate MulTI-Tag for H3K27me3 and PolIIS5P in K562 cells, sequentially profiled
MM_Hs_MPM1075Ser5; K562_Ser5_MulTI_secondary_seq; PolIIS5P-barcoded data from secondary antibody conjugate MulTI-Tag for H3K27me3 and PolIIS5P in K562 cells, sequentially profiled
MM_Hs_MPM1076K27; K562_K27_MulTI_secondary_comb; H3K27me3-barcoded data from secconday antibody conjugate MulTI-Tag for H3K27me3 and PolIIS5P in K562 cells, simultaneously profiled
MM_Hs_MPM1076Ser5; K562_Ser5_MulTI_secondary_comb; PolIIS5P-barcoded data from secondary antibody conjugate MulTI-Tag for H3K27me3 and PolIIS5P in K562 cells, simultaneously profiled
MM_Hs_MPM1487; K562_K27_MulTI_primary_indiv_rep1; Primary antibody conjugate MulTI-Tag for H3K27me3 in K562 cells, individually profiled
MM_Hs_MPM1488; K562_Ser5_MulTI_primary_indiv_rep1; Primary antibody conjugate MulTI-Tag for PolIIS5P in K562 cells, individually profiled
MM_Hs_MPM1489K27; K562_K27_MulTI_primary_seq_rep1; H3K27me3-barcoded data from primary antibody conjugate MulTI-Tag for H3K27me3 and PolIIS5P in K562 cells, sequentially profiled
MM_Hs_MPM1489Ser5; K562_Ser5_MulTI_primary_seq_rep1; PolIIS5P-barcoded data from primary antibody conjugate MulTI-Tag for H3K27me3 and PolIIS5P in K562 cells, sequentially profiled
MM_Hs_MPM1490K27; K562_K27_MulTI_primary_comb; H3K27me3-barcoded data from primary antibody conjugate MulTI-Tag for H3K27me3 and PolIIS5P in K562 cells, simultaneously profiled
MM_Hs_MPM1490Ser5; K562_Ser5_MulTI_primary_comb; PolIIS5P-barcoded data from primary antibody conjugate MulTI-Tag for H3K27me3 and PolIIS5P in K562 cells, simultaneously profiled
MM_Hs_MPM1527; K562_K27_MulTI_primary_indiv_rep2; Primary antibody conjugate MulTI-Tag for H3K27me3 in K562 cells, individually profiled
MM_Hs_MPM1528; K562_Ser5_MulTI_primary_indiv_rep2; Primary antibody conjugate MulTI-Tag for PolIIS5P in K562 cells, individually profiled
MM_Hs_MPM1531K27; K562_K27_MulTI_primary_seq_rep2; H3K27me3-barcoded data from primary antibody conjugate MulTI-Tag for H3K27me3 and PolIIS5P in K562 cells, sequentially profiled
MM_Hs_MPM1531Ser5; K562_Ser5_MulTI_primary_seq_rep2; PolIIS5P-barcoded data from primary antibody conjugate MulTI-Tag for H3K27me3 and PolIIS5P in K562 cells, sequentially profiled
MM_Hs_MPM1953; K562_K27_MulTI_indiv; MulTI-Tag for H3K27me3 in K562 cells, individually profiled
MM_Hs_MPM1954; K562_K4_MulTI_indiv; MulTI-Tag for H3K4me2 in K562 cells, individually profiled
MM_Hs_MPM1955; K562_K36_MulTI_indiv; MulTI-Tag for H3K36me3 in K562 cells, individually profiled
MM_Hs_MPM1956K27; K562_K27_MulTI_seq; H3K27me3-barcoded data from MulTI-Tag for H3K27me3, H3K4me2, and H3K36me3 in K562 cells, sequentially profiled
MM_Hs_MPM1956K4; K562_K4_MulTI_seq; H3K4me2-barcoded data from MulTI-Tag for H3K27me3, H3K4me2, and H3K36me3 in K562 cells, sequentially profiled
MM_Hs_MPM1956K36; K562_K36_MulTI_seq; H3K36me3-barcoded data from MulTI-Tag for H3K27me3, H3K4me2, and H3K36me3 in K562 cells, sequentially profiled

MM_Hs_MPM1957; H1_K27_MulTI_indiv; MulTI-Tag for H3K27me3 in H1 cells, individually profiled
MM_Hs_MPM1958; H1_K4_MulTI_indiv; MulTI-Tag for H3K4me2 in H1 cells, individually profiled
MM_Hs_MPM1959; H1_K36_MulTI_indiv; MulTI-Tag for H3K36me3 in H1 cells, individually profiled
MM_Hs_MPM1960K27; H1_K27_MulTI_seq; H3K27me3-barcoded data from MulTI-Tag for H3K27me3, H3K4me2, and H3K36me3 in H1 cells, sequentially profiled
MM_Hs_MPM1960K4; H1_K4_MulTI_seq; H3K4me2-barcoded data from MulTI-Tag for H3K27me3, H3K4me2, and H3K36me3 in H1 cells, sequentially profiled
MM_Hs_MPM1960K36; H1_K36_MulTI_seq; H3K36me3-barcoded data from MulTI-Tag for H3K27me3, H3K4me2, and H3K36me3 in H1 cells, sequentially profiled
MM_Hs_MPM1961; K562_K27_MulTI_indiv_ICELL8_rep1; MulTI-Tag for H3K27me3 in K562 cells, individually profiled, amplified on ICELL8
MM_Hs_MPM1962; K562_K4_MulTI_indiv_ICELL8_rep1; MulTI-Tag for H3K4me2 in K562 cells, individually profiled, amplified on ICELL8
MM_Hs_MPM1963; K562_K36_MulTI_indiv_ICELL8_rep1; MulTI-Tag for H3K36me3 in K562 cells, individually profiled, amplified on ICELL8
MM_Hs_MPM1964K27; K562_K27_MulTI_seq_ICELL8_rep1; H3K27me3-barcoded data from MulTI-Tag for H3K27me3, H3K4me2, and H3K36me3 in K562 cells, sequentially profiled, amplified on ICELL8
MM_Hs_MPM1964K4; K562_K4_MulTI_seq_ICELL8_rep1; H3K4me2-barcoded data from MulTI-Tag for H3K27me3, H3K4me2, and H3K36me3 in K562 cells, sequentially profiled, amplified on ICELL8
MM_Hs_MPM1964K36; K562_K36_MulTI_seq_ICELL8_rep1; H3K36me3-barcoded data from MulTI-Tag for H3K27me3, H3K4me2, and H3K36me3 in K562 cells, sequentially profiled, amplified on ICELL8
MM_HsMm_1989-93; K562-3T3_K27_MulTI_indiv_ICELL8_rep1; MulTI-Tag for H3K27me3 in a mixture of K562 and NIH3T3 cells, individually profiled, amplified on ICELL8
MM_HsMm_1990-94; K562-3T3_K4_MulTI_indiv_ICELL8_rep1; MulTI-Tag for H3K4me2 in a mixture of K562 and NIH3T3 cells, individually profiled, amplified on ICELL8
MM_HsMm_1991-95; K562-3T3_K36_MulTI_indiv_ICELL8_rep1; MulTI-Tag for H3K36me3 in a mixture of K562 and NIH3T3 cells, individually profiled, amplified on ICELL8
MM_HsMm_1992-96K27; K562-3T3_K27_MulTI_seq_ICELL8_rep1; H3K27me3-barcoded data from MulTI-Tag for H3K27me3, H3K4me2, and H3K36me3 in a mixture of K562 and NIH3T3 cells, sequentially profiled, amplified on ICELL8
MM_HsMm_1992-96K4; K562-3T3_K4_MulTI_seq_ICELL8_rep1; H3K4me2-barcoded data from MulTI-Tag for H3K27me3, H3K4me2, and H3K36me3 in a mixture of K562 and NIH3T3 cells, sequentially profiled, amplified on ICELL8
MM_HsMm_1992-96K36; K562-3T3_K36_MulTI_seq_ICELL8_rep1; H3K36me3-barcoded data from MulTI-Tag for H3K27me3, H3K4me2, and H3K36me3 in a mixture of K562 and NIH3T3 cells , sequentially profiled, amplified on ICELL8
MM_Hs_MPM2057; K562_K27_MulTI_indiv_ICELL8_rep2; MulTI-Tag for H3K27me3 in K562 cells, individually profiled, amplified on ICELL8
MM_Hs_MPM2058; K562_K4_MulTI_indiv_ICELL8_rep2; MulTI-Tag for H3K4me2 in K562 cells, individually profiled, amplified on ICELL8
MM_Hs_MPM2059; K562_K36_MulTI_indiv_ICELL8_rep2; MulTI-Tag for H3K36me3 in K562 cells, individually profiled, amplified on ICELL8
MM_Hs_MPM2060K27; K562_K27_MulTI_seq_ICELL8_rep2; H3K27me3-barcoded data from MulTI-Tag for H3K27me3, H3K4me2, and H3K36me3 in K562 cells, sequentially profiled, amplified on ICELL8
MM_Hs_MPM2060K4; K562_K4_MulTI_seq_ICELL8_rep2; H3K4me2-barcoded data from MulTI-Tag for H3K27me3, H3K4me2, and H3K36me3 in K562 cells, sequentially profiled, amplified on ICELL8
MM_Hs_MPM2060K36; K562_K36_MulTI_seq_ICELL8_rep2; H3K36me3-barcoded data from MulTI-Tag for H3K27me3, H3K4me2, and H3K36me3 in K562 cells, sequentially profiled, amplified on ICELL8
MM_Hs_MPM2244K27; K562_K27_MulTI_seq_ICELL8; H3K27me3-barcoded data from MulTI-Tag for H3K27me3, H3K4me2, and H3K36me3 in K562 cells, sequentially profiled, amplified on ICELL8
MM_Hs_MPM2244K4; K562_K4_MulTI_seq_ICELL8; H3K4me2-barcoded data from MulTI-Tag for H3K27me3, H3K4me2, and H3K36me3 in K562 cells, sequentially profiled, amplified on ICELL8
MM_Hs_MPM2244K36; K562_K36_MulTI_seq_ICELL8; H3K36me3-barcoded data from MulTI-Tag for H3K27me3, H3K4me2, and H3K36me3 in K562 cells, sequentially profiled, amplified on ICELL8
MM_Hs_MPM2246K27; H1_K27_MulTI_seq_ICELL8; H3K27me3-barcoded data from MulTI-Tag for H3K27me3, H3K4me2, and H3K36me3 in H1 cells, sequentially profiled, amplified on ICELL8
MM_Hs_MPM2246K4; H1_K4_MulTI_seq_ICELL8; H3K4me2-barcoded data from MulTI-Tag for H3K27me3, H3K4me2, and H3K36me3 in H1 cells, sequentially profiled, amplified on ICELL8
MM_Hs_MPM2246K36; H1_K36_MulTI_seq_ICELL8; H3K36me3-barcoded data from MulTI-Tag for H3K27me3, H3K4me2, and H3K36me3 in H1 cells, sequentially profiled, amplified on ICELL8
MM_Hs_MPM2350K27; K562_K27_MulTI_seq_ICELL8_2; H3K27me3-barcoded data from MulTI-Tag for H3K27me3 and H3K36me3 in K562 cells, sequentially profiled, amplified on ICELL8
MM_Hs_MPM2350K36; K562_K36_MulTI_seq_ICELL8_2; H3K36me3-barcoded data from MulTI-Tag for H3K27me3 and H3K36me3 in K562 cells, sequentially profiled, amplified on ICELL8
MM_Hs_MPM2351K27; H1_K27_MulTI_seq_ICELL8_2; H3K27me3-barcoded data from MulTI-Tag for H3K27me3 and H3K36me3 in H1 cells, sequentially profiled, amplified on ICELL8
MM_Hs_MPM2351K36; H1_K36_MulTI_seq_ICELL8_2; H3K36me3-barcoded data from MulTI-Tag for H3K27me3 and H3K36me3 in H1 cells, sequentially profiled, amplified on ICELL8
MM_Hs_MPM2352K27; K562-H1_K27_MulTI_seq_ICELL8_2; H3K27me3-barcoded data from MulTI-Tag for H3K27me3 and H3K36me3 in a mix of K562 and H1 cells, sequentially profiled, amplified on ICELL8
MM_Hs_MPM2352K36; K562-H1_K36_MulTI_seq_ICELL8_2; H3K36me3-barcoded data from MulTI-Tag for H3K27me3 and H3K36me3 in a mix of K562 and H1 cells, sequentially profiled, amplified on ICELL8
MM_Hs_MPM2545; K562-3T3_K27_MulTI_indiv_ICELL8; H3K27me3-barcoded data from MulTI-Tag for H3K27me3 in a mix of K562 and NIH3T3 cells, amplified on ICELL8
MM_Hs_MPM2546; K562-3T3_K36_MulTI_indiv_ICELL8; H3K36me3-barcoded data from MulTI-Tag for H3K36me3 in a mix of K562 and NIH3T3 cells, amplified on ICELL8
MM_Hs_MPM2547K27; K562-3T3_K27_MulTI_seq_ICELL8; H3K27me3-barcoded data from MulTI-Tag for H3K27me3 and H3K36me3 in a mix of K562 and NIH3T3 cells, sequentially profiled, amplified on ICELL8
MM_Hs_MPM2547K27; K562-3T3_K36_MulTI_seq_ICELL8; H3K36me3-barcoded data from MulTI-Tag for H3K27me3 and

H3K36me3 in a mix of K562 and NIH3T3 cells, sequentially profiled, amplified on ICELL8
MM_Hs_MPM2581K27; K562_K27_MulTI_seq_ICELL8_3; H3K27me3-barcoded data from MulTI-Tag for H3K27me3 and PolIIS5P in K562 cells, sequentially profiled, amplified on ICELL8
MM_Hs_MPM2581Ser5; K562_Ser5_MulTI_seq_ICELL8_3; PolIIS5P-barcoded data from MulTI-Tag for H3K27me3 and PolIIS5P in K562 cells, sequentially profiled, amplified on ICELL8
MM_Hs_MPM2582K27; H1_K27_MulTI_seq_ICELL8_3; H3K27me3-barcoded data from MulTI-Tag for H3K27me3 and PolIIS5P in H1 cells, sequentially profiled, amplified on ICELL8
MM_Hs_MPM2582Ser5; H1_Ser5_MulTI_seq_ICELL8_3; PolIIS5P-barcoded data from MulTI-Tag for H3K27me3 and PolIIS5P in H1 cells, sequentially profiled, amplified on ICELL8
MM_Hs_MPM2583K27; K562-H1_K27_MulTI_seq_ICELL8_3; H3K27me3-barcoded data from MulTI-Tag for H3K27me3 and PolIIS5P in a mix of K562 and H1 cells, sequentially profiled, amplified on ICELL8
MM_Hs_MPM2583Ser5; K562-H1_Ser5_MulTI_seq_ICELL8_3; PolIIS5P-barcoded data from MulTI-Tag for H3K27me3 and PolIIS5P in a mix of K562 and H1 cells, sequentially profiled, amplified on ICELL8
MM_Hs_MPM2584K27; K562_K27_MulTI_seq_ICELL8_4; H3K27me3-barcoded data from MulTI-Tag for H3K27me3 and H3K9me3 in K562 cells, sequentially profiled, amplified on ICELL8
MM_Hs_MPM2584K9; K562_K9_MulTI_seq_ICELL8_4; H3K9me3-barcoded data from MulTI-Tag for H3K27me3 and H3K9me3 in K562 cells, sequentially profiled, amplified on ICELL8
MM_Hs_MPM2585K27; H1_K27_MulTI_seq_ICELL8_4; H3K27me3-barcoded data from MulTI-Tag for H3K27me3 and H3K9me3 in H1 cells, sequentially profiled, amplified on ICELL8
MM_Hs_MPM2585K9; H1_K9_MulTI_seq_ICELL8_4; H3K9me3-barcoded data from MulTI-Tag for H3K27me3 and H3K9me3 in H1 cells, sequentially profiled, amplified on ICELL8
MM_Hs_MPM2586K27; K562-H1_K27_MulTI_seq_ICELL8_4; H3K27me3-barcoded data from MulTI-Tag for H3K27me3 and H3K9me3 in a mix of K562 and H1 cells, sequentially profiled, amplified on ICELL8
MM_Hs_MPM2586K9; K562-H1_K9_MulTI_seq_ICELL8_4; H3K9me3-barcoded data from MulTI-Tag for H3K27me3 and H3K9me3 in a mix of K562 and H1 cells, sequentially profiled, amplified on ICELL8
MM_Hs_MPM2587K27; K562_K27_MulTI_seq_ICELL8_5; H3K27me3-barcoded data from MulTI-Tag for H3K27me3 and H3K4me1 in K562 cells, sequentially profiled, amplified on ICELL8
MM_Hs_MPM2587K4m1; K562_K4m1_MulTI_seq_ICELL8_5; H3K4me1-barcoded data from MulTI-Tag for H3K27me3 and H3K4me1 in K562 cells, sequentially profiled, amplified on ICELL8
MM_Hs_MPM2588K27; H1_K27_MulTI_seq_ICELL8_5; H3K27me3-barcoded data from MulTI-Tag for H3K27me3 and H3K4me1 in H1 cells, sequentially profiled, amplified on ICELL8
MM_Hs_MPM2588K4m1; H1_K4m1_MulTI_seq_ICELL8_5; H3K4me1-barcoded data from MulTI-Tag for H3K27me3 and H3K4me1 in H1 cells, sequentially profiled, amplified on ICELL8
MM_Hs_MPM2589K27; K562-H1_K27_MulTI_seq_ICELL8_5; H3K27me3-barcoded data from MulTI-Tag for H3K27me3 and H3K4me1 in a mix of K562 and H1 cells, sequentially profiled, amplified on ICELL8
MM_Hs_MPM2589K4m1; K562-H1_K4m1_MulTI_seq_ICELL8_5; H3K4me1-barcoded data from MulTI-Tag for H3K27me3 and H3K4me1 in a mix of K562 and H1 cells, sequentially profiled, amplified on ICELL8
MM_Hs_MPM2590K27; K562_K27_MulTI_seq_ICELL8_6; H3K27me3-barcoded data from MulTI-Tag for H3K27me3 and H3K36me3 in K562 cells, sequentially profiled, amplified on ICELL8
MM_Hs_MPM2590K27; K562_K36_MulTI_seq_ICELL8_6; H3K36me3-barcoded data from MulTI-Tag for H3K27me3 and H3K36me3 in K562 cells, sequentially profiled, amplified on ICELL8
MM_Hs_MPM2591K27; H1_K27_MulTI_seq_ICELL8_6; H3K27me3-barcoded data from MulTI-Tag for H3K27me3 and H3K36me3 in H1 cells, sequentially profiled, amplified on ICELL8
MM_Hs_MPM2591K27; H1_K36_MulTI_seq_ICELL8_6; H3K36me3-barcoded data from MulTI-Tag for H3K27me3 and H3K36me3 in H1 cells, sequentially profiled, amplified on ICELL8
MM_Hs_MPM2592K27; K562-H1_K27_MulTI_seq_ICELL8_6; H3K27me3-barcoded data from MulTI-Tag for H3K27me3 and H3K36me3 in a mix of K562 and H1 cells, sequentially profiled, amplified on ICELL8
MM_Hs_MPM2592K27; K562-H1_K36_MulTI_seq_ICELL8_6; H3K36me3-barcoded data from MulTI-Tag for H3K27me3 and H3K36me3 in a mix of K562 and H1 cells, sequentially profiled, amplified on ICELL8
MM_Hs_MPM2611K27; hESC_K27_MulTI_seq_ICELL8; H3K27me3-barcoded data from MulTI-Tag for H3K27me3, H3K4me1, and H3K36me3 in H1 cells, sequentially profiled, amplified on ICELL8
MM_Hs_MPM2611K4m1; hESC_K4m1_MulTI_seq_ICELL8; H3K4me1-barcoded data from MulTI-Tag for H3K27me3, H3K4me1, and H3K36me3 in H1 cells, sequentially profiled, amplified on ICELL8
MM_Hs_MPM2611K36; hESC_K36_MulTI_seq_ICELL8; H3K36me3-barcoded data from MulTI-Tag for H3K27me3, H3K4me1, and H3K36me3 in H1 cells, sequentially profiled, amplified on ICELL8
MM_Hs_MPM2612K27; DE2_K27_MulTI_seq_ICELL8; H3K27me3-barcoded data from MulTI-Tag for H3K27me3, H3K4me1, and H3K36me3 in Day 2 Endoderm cells, sequentially profiled, amplified on ICELL8
MM_Hs_MPM2612K4m1; DE2_K4m1_MulTI_seq_ICELL8; H3K4me1-barcoded data from MulTI-Tag for H3K27me3, H3K4me1, and H3K36me3 in Day 2 Endoderm cells, sequentially profiled, amplified on ICELL8
MM_Hs_MPM2612K36; DE2_K36_MulTI_seq_ICELL8; H3K36me3-barcoded data from MulTI-Tag for H3K27me3, H3K4me1, and H3K36me3 in Day 2 Endoderm cells, sequentially profiled, amplified on ICELL8
MM_Hs_MPM2613K27; DE3_K27_MulTI_seq_ICELL8; H3K27me3-barcoded data from MulTI-Tag for H3K27me3, H3K4me1, and H3K36me3 in Day 3 Endoderm cells, sequentially profiled, amplified on ICELL8
MM_Hs_MPM2613K4m1; DE3_K4m1_MulTI_seq_ICELL8; H3K4me1-barcoded data from MulTI-Tag for H3K27me3, H3K4me1, and H3K36me3 in Day 3 Endoderm cells, sequentially profiled, amplified on ICELL8
MM_Hs_MPM2613K36; DE3_K36_MulTI_seq_ICELL8; H3K36me3-barcoded data from MulTI-Tag for H3K27me3, H3K4me1, and H3K36me3 in Day 3 Endoderm cells, sequentially profiled, amplified on ICELL8
MM_Hs_MPM2614K27; DE4_K27_MulTI_seq_ICELL8; H3K27me3-barcoded data from MulTI-Tag for H3K27me3, H3K4me1, and H3K36me3 in Day 4 Endoderm cells, sequentially profiled, amplified on ICELL8
MM_Hs_MPM2614K4m1; DE4_K4m1_MulTI_seq_ICELL8; H3K4me1-barcoded data from MulTI-Tag for H3K27me3, H3K4me1, and H3K36me3 in Day 4 Endoderm cells, sequentially profiled, amplified on ICELL8
MM_Hs_MPM2614K36; DE4_K36_MulTI_seq_ICELL8; H3K36me3-barcoded data from MulTI-Tag for H3K27me3, H3K4me1, and H3K36me3 in Day 4 Endoderm cells, sequentially profiled, amplified on ICELL8
MM_Hs_MPM2615K27; DE5_K27_MulTI_seq_ICELL8; H3K27me3-barcoded data from MulTI-Tag for H3K27me3, H3K4me1, and H3K36me3 in Day 5 Endoderm cells, sequentially profiled, amplified on ICELL8
MM_Hs_MPM2615K4m1; DE5_K4m1_MulTI_seq_ICELL8; H3K4me1-barcoded data from MulTI-Tag for H3K27me3,

H3K4me1, and H3K36me3 in Day 5 Endoderm cells, sequentially profiled, amplified on ICELL8
MM_Hs_MPM2615K36; DE5_K36_MulTI_seq_ICELL8; H3K36me3-barcoded data from MulTI-Tag for H3K27me3, H3K4me1, and H3K36me3 in Day 5 Endoderm cells, sequentially profiled, amplified on ICELL8
MM_Hs_MPM2616K27; MES2_K27_MulTI_seq_ICELL8; H3K27me3-barcoded data from MulTI-Tag for H3K27me3, H3K4me1, and H3K36me3 in Day 2 Mesoderm cells, sequentially profiled, amplified on ICELL8
MM_Hs_MPM2616K4m1; MES2_K4m1_MulTI_seq_ICELL8; H3K4me1-barcoded data from MulTI-Tag for H3K27me3, H3K4me1, and H3K36me3 in Day 2 Mesoderm cells, sequentially profiled, amplified on ICELL8
MM_Hs_MPM2616K36; MES2_K36_MulTI_seq_ICELL8; H3K36me3-barcoded data from MulTI-Tag for H3K27me3, H3K4me1, and H3K36me3 in Day 2 Mesoderm cells, sequentially profiled, amplified on ICELL8
MM_Hs_MPM2617K27; MES3_K27_MulTI_seq_ICELL8; H3K27me3-barcoded data from MulTI-Tag for H3K27me3, H3K4me1, and H3K36me3 in Day 3 Mesoderm cells, sequentially profiled, amplified on ICELL8
MM_Hs_MPM2617K4m1; MES3_K4m1_MulTI_seq_ICELL8; H3K4me1-barcoded data from MulTI-Tag for H3K27me3, H3K4me1, and H3K36me3 in Day 3 Mesoderm cells, sequentially profiled, amplified on ICELL8
MM_Hs_MPM2617K36; MES3_K36_MulTI_seq_ICELL8; H3K36me3-barcoded data from MulTI-Tag for H3K27me3, H3K4me1, and H3K36me3 in Day 3 Mesoderm cells, sequentially profiled, amplified on ICELL8
MM_Hs_MPM2618K27; MES4_K27_MulTI_seq_ICELL8; H3K27me3-barcoded data from MulTI-Tag for H3K27me3, H3K4me1, and H3K36me3 in Day 4 Mesoderm cells, sequentially profiled, amplified on ICELL8
MM_Hs_MPM2618K4m1; MES4_K4m1_MulTI_seq_ICELL8; H3K4me1-barcoded data from MulTI-Tag for H3K27me3, H3K4me1, and H3K36me3 in Day 4 Mesoderm cells, sequentially profiled, amplified on ICELL8
MM_Hs_MPM2618K36; MES4_K36_MulTI_seq_ICELL8; H3K36me3-barcoded data from MulTI-Tag for H3K27me3, H3K4me1, and H3K36me3 in Day 4 Mesoderm cells, sequentially profiled, amplified on ICELL8
MM_Hs_MPM2619K27; MES5_K27_MulTI_seq_ICELL8; H3K27me3-barcoded data from MulTI-Tag for H3K27me3, H3K4me1, and H3K36me3 in Day 5 Mesoderm cells, sequentially profiled, amplified on ICELL8
MM_Hs_MPM2619K4m1; MES5_K4m1_MulTI_seq_ICELL8; H3K4me1-barcoded data from MulTI-Tag for H3K27me3, H3K4me1, and H3K36me3 in Day 5 Mesoderm cells, sequentially profiled, amplified on ICELL8
MM_Hs_MPM2619K36; MES5_K36_MulTI_seq_ICELL8; H3K36me3-barcoded data from MulTI-Tag for H3K27me3, H3K4me1, and H3K36me3 in Day 5 Mesoderm cells, sequentially profiled, amplified on ICELL8
MM_Hs_MPM2620K27; ECT2_K27_MulTI_seq_ICELL8; H3K27me3-barcoded data from MulTI-Tag for H3K27me3, H3K4me1, and H3K36me3 in Day 2 Ectoderm cells, sequentially profiled, amplified on ICELL8
MM_Hs_MPM2620K4m1; ECT2_K4m1_MulTI_seq_ICELL8; H3K4me1-barcoded data from MulTI-Tag for H3K27me3, H3K4me1, and H3K36me3 in Day 2 Ectoderm cells, sequentially profiled, amplified on ICELL8
MM_Hs_MPM2620K36; ECT2_K36_MulTI_seq_ICELL8; H3K36me3-barcoded data from MulTI-Tag for H3K27me3, H3K4me1, and H3K36me3 in Day 2 Ectoderm cells, sequentially profiled, amplified on ICELL8
MM_Hs_MPM2621K27; ECT3_K27_MulTI_seq_ICELL8; H3K27me3-barcoded data from MulTI-Tag for H3K27me3, H3K4me1, and H3K36me3 in Day 3 Ectoderm cells, sequentially profiled, amplified on ICELL8
MM_Hs_MPM2621K4m1; ECT3_K4m1_MulTI_seq_ICELL8; H3K4me1-barcoded data from MulTI-Tag for H3K27me3, H3K4me1, and H3K36me3 in Day 3 Ectoderm cells, sequentially profiled, amplified on ICELL8
MM_Hs_MPM2621K36; ECT3_K36_MulTI_seq_ICELL8; H3K36me3-barcoded data from MulTI-Tag for H3K27me3, H3K4me1, and H3K36me3 in Day 3 Ectoderm cells, sequentially profiled, amplified on ICELL8
MM_Hs_MPM2622K27; ECT4_K27_MulTI_seq_ICELL8; H3K27me3-barcoded data from MulTI-Tag for H3K27me3, H3K4me1, and H3K36me3 in Day 4 Ectoderm cells, sequentially profiled, amplified on ICELL8
MM_Hs_MPM2622K4m1; ECT4_K4m1_MulTI_seq_ICELL8; H3K4me1-barcoded data from MulTI-Tag for H3K27me3, H3K4me1, and H3K36me3 in Day 4 Ectoderm cells, sequentially profiled, amplified on ICELL8
MM_Hs_MPM2622K36; ECT4_K36_MulTI_seq_ICELL8; H3K36me3-barcoded data from MulTI-Tag for H3K27me3, H3K4me1, and H3K36me3 in Day 4 Ectoderm cells, sequentially profiled, amplified on ICELL8
MM_Hs_MPM2623K27; ECT5_K27_MulTI_seq_ICELL8; H3K27me3-barcoded data from MulTI-Tag for H3K27me3, H3K4me1, and H3K36me3 in Day 5 Ectoderm cells, sequentially profiled, amplified on ICELL8
MM_Hs_MPM2623K4m1; ECT5_K4m1_MulTI_seq_ICELL8; H3K4me1-barcoded data from MulTI-Tag for H3K27me3, H3K4me1, and H3K36me3 in Day 5 Ectoderm cells, sequentially profiled, amplified on ICELL8
MM_Hs_MPM2623K36; ECT5_K36_MulTI_seq_ICELL8; H3K36me3-barcoded data from MulTI-Tag for H3K27me3, H3K4me1, and H3K36me3 in Day 5 Ectoderm cells, sequentially profiled, amplified on ICELL8
MM_Hs_MPM2624K27; ECT6_K27_MulTI_seq_ICELL8; H3K27me3-barcoded data from MulTI-Tag for H3K27me3, H3K4me1, and H3K36me3 in Day 6 Ectoderm cells, sequentially profiled, amplified on ICELL8
MM_Hs_MPM2624K4m1; ECT6_K4m1_MulTI_seq_ICELL8; H3K4me1-barcoded data from MulTI-Tag for H3K27me3, H3K4me1, and H3K36me3 in Day 6 Ectoderm cells, sequentially profiled, amplified on ICELL8
MM_Hs_MPM2624K36; ECT6_K36_MulTI_seq_ICELL8; H3K36me3-barcoded data from MulTI-Tag for H3K27me3, H3K4me1, and H3K36me3 in Day 6 Ectoderm cells, sequentially profiled, amplified on ICELL8
MM_Hs_MPM2625K27; ECT7_K27_MulTI_seq_ICELL8; H3K27me3-barcoded data from MulTI-Tag for H3K27me3, H3K4me1, and H3K36me3 in Day 7 Ectoderm cells, sequentially profiled, amplified on ICELL8
MM_Hs_MPM2625K4m1; ECT7_K4m1_MulTI_seq_ICELL8; H3K4me1-barcoded data from MulTI-Tag for H3K27me3, H3K4me1, and H3K36me3 in Day 7 Ectoderm cells, sequentially profiled, amplified on ICELL8
MM_Hs_MPM2625K36; ECT7_K36_MulTI_seq_ICELL8; H3K36me3-barcoded data from MulTI-Tag for H3K27me3, H3K4me1, and H3K36me3 in Day 7 Ectoderm cells, sequentially profiled, amplified on ICELL8

Genome browser session
(e.g. UCSC)

No longer applicable.

Methodology

Replicates

Replication is described in Supplementary Table 2.

Sequencing depth

All experiments were paired end. Sequencing depths and statistics are provided in Supplementary Table 2.

Antibodies

Antibodies used for CUT&Tag/MulTI-Tag: Rabbit anti-H3K27me3 (Cell Signaling Technologies CST 9733, Lot 16), Mouse anti-RNA

Antibodies	PolIIS5P (Abcam ab5408, Lot GR3264297-2), Mouse anti-H3K4me2 (Active Motif 39679, Lot 31718013), Mouse anti-H3K36me3 (Active Motif 61021, Lot 23819012), Rabbit anti-H3K9me3 (Abcam ab8898, Lot GR3302452-1), Rabbit anti-H3K4me1 (EpiCypher 13-0040, Lot 2134006-02), Guinea Pig anti-Rabbit (Antibodies Online ABIN101961), Rabbit anti-Mouse (Abcam ab46450).
Peak calling parameters	SEACRv1.4, as described in Methods
Data quality	Data quality was evaluated by alignment rate (listed for all datasets in Supplementary Table 2), and by cross-mapping between MulTI-Tag signal for one target to non-target peaks as described in text.
Software	All code necessary for analysis presented in this study are provided at doi.org://10.5281/zenodo.6636675
